# A combined model reduction algorithm for controlled biochemical systems

**DOI:** 10.1186/s12918-017-0397-1

**Published:** 2017-02-13

**Authors:** Thomas J. Snowden, Piet H. van der Graaf, Marcus J. Tindall

**Affiliations:** 1 Department of Mathematics and Statistics, University of Reading, Reading, RG6 6AX UK; 2 Certara QSP, University of Kent Innovation Centre, Canterbury, CT2 7FG UK; 3 Leiden Academic Centre for Drug Research, Universiteit Leiden, Leiden, NL-2333 CC Netherlands; 4 The Institute for Cardiovascular and Metabolic Research (ICMR), University of Reading, Reading, RG6 6AX UK

**Keywords:** Model reduction, Lumping, Empirical balanced truncation, Controlled systems

## Abstract

**Background:**

Systems Biology continues to produce increasingly large models of complex biochemical reaction networks. In applications requiring, for example, parameter estimation, the use of agent-based modelling approaches, or real-time simulation, this growing model complexity can present a significant hurdle. Often, however, not all portions of a model are of equal interest in a given setting. In such situations methods of model reduction offer one possible approach for addressing the issue of complexity by seeking to eliminate those portions of a pathway that can be shown to have the least effect upon the properties of interest.

**Methods:**

In this paper a model reduction algorithm bringing together the complementary aspects of proper lumping and empirical balanced truncation is presented. Additional contributions include the development of a criterion for the selection of state-variable elimination via conservation analysis and use of an ‘averaged’ lumping inverse. This combined algorithm is highly automatable and of particular applicability in the context of ‘controlled’ biochemical networks.

**Results:**

The algorithm is demonstrated here via application to two examples; an 11 dimensional model of bacterial chemotaxis in *Escherichia coli* and a 99 dimensional model of extracellular regulatory kinase activation (ERK) mediated via the epidermal growth factor (EGF) and nerve growth factor (NGF) receptor pathways. In the case of the chemotaxis model the algorithm was able to reduce the model to 2 state-variables producing a maximal relative error between the dynamics of the original and reduced models of only 2.8*%* whilst yielding a 26 fold speed up in simulation time. For the ERK activation model the algorithm was able to reduce the system to 7 state-variables, incurring a maximal relative error of 4.8*%*, and producing an approximately 10 fold speed up in the rate of simulation. Indices of controllability and observability are additionally developed and demonstrated throughout the paper. These provide insight into the relative importance of individual reactants in mediating a biochemical system’s input-output response even for highly complex networks.

**Conclusions:**

Through application, this paper demonstrates that combined model reduction methods can produce a significant simplification of complex Systems Biology models whilst retaining a high degree of predictive accuracy. In particular, it is shown that by combining the methods of proper lumping and empirical balanced truncation it is often possible to produce more accurate reductions than can be obtained by the use of either method in isolation.

**Electronic supplementary material:**

The online version of this article (doi:10.1186/s12918-017-0397-1) contains supplementary material, which is available to authorized users.

## Background

The field of Systems Biology has seen a considerable increase in both the number of models created and their complexity across the past decade. The BioModels Database, which acts as an open repository for Systems Biology models, saw the number of models it stores increase approximately ten-fold between 2005 and 2010, with the average number of reactions per model having nearly tripled in the same period [1]. This increase in complexity, specifically in the number of species or reactions modelled by each system, has become a defining characteristic of research in this area.

Such systems are typically developed by bringing together biochemical and physiological knowledge to inform highly detailed mechanistic models of biological networks (e.g. signalling pathways, protein-protein interactions, and genetic cascades). Mathematically, these networks are typically modelled via high-dimensional systems of stiff, nonlinear ordinary differential equations (ODEs).

This model complexity, however, can present a number of issues with regards to their use and analysis. For example parameter estimation techniques and real-time numerical simulation can both be difficult to perform for high dimensional or overparameterised systems. Even the basic intuitiveness of a system can potentially be obscured by its complexity. Additionally, complexity of this form is often associated with the ‘curse of dimensionality’, whereby the data that can be obtained for such systems in practice are sparse relative to the volume of the state and parameter spaces.

Model reduction techniques [2] offer one possible approach to easing complexity. A method of model reduction here refers to any method designed to construct a lower order representation (either in terms of the number of state variables or parameters) of a model with which some set of the original dynamical behaviour can be satisfactorily approximated.

A range of model reduction methods exist in the literature, many of which have previously been applied to models of biochemical reaction networks. The most commonly applied methods are based upon time-scale separation. These simplify a system by exploiting the wide ranges in reaction rates and equilibration speeds typical of biochemical reaction networks. These approaches include variants of the quasi-steady state approximation [3–9], variants of the rapid equilibrium approximation [10–15], the intrinsic low dimensional manifold method [16–20], and computational singular perturbation [21–24]. Beyond time-scale exploitation, sensitivity analysis can also be used to guide model reduction by identifying and eliminating those portions of a network least responsible for the dynamical behaviour of interest [25–29]. Optimisation based methods [30–35] seek to evaluate a range of possible reduced models under a given metric of reduction accuracy before returning the best available option. Lumping methods, meanwhile, reduce a network by treating groups of state-variables as a single dynamical, ‘lumped’ variable [36–41]. Additionally, there exist a range of singular value decomposition (SVD) reduction methods based upon the matrix decomposition of the same name. These exploit the property that SVD can be used to give lower rank approximations of matrices. Such methods have seen limited application to biochemical systems, but a number of publications employing a particular variant known as balanced truncation can be found in the literature [42–44].

Each model reduction approach has advantages and disadvantages in the reduction of large scale models of biochemical reaction networks. There is no ‘one size fits all’ method of reduction; the best method available depends inextricably on the properties of the model and the aims of reduction.

Systems Biology models, such as those this paper seeks to reduce, usually possess a number of mathematical properties that can influence the suitability of specific reduction methodologies: most notably, such models are often stiff, nonlinear and of extremely high dimensionality (e.g. containing 10s or even 100s of state-variables and parameters). Stiffness, in this context, refers to a parameter dependent property of a system of differential equations whereby their numerical integration can require taking a step-length that is excessively short relative to the exact solution’s smoothness in a given interval. This has relatively severe implications for simulating such models, as stiffness is associated with issues of numerical stability and computational processing time. In the case of large-scale Systems Biology models, stiffness is typically a consequence of reactions in the system evolving and equilibrating at greatly different timescales as compared with one another. Meanwhile, the nonlinearity of these models implies that a number of analytical methodologies will not be applicable. Often linearisation is used in this context, but in many such cases this incurs a prohibitively large error. Finally, the high dimensionality of such systems can also present issues of combinatorial complexity or an excessive computational burden for some methods of mathematical analysis.

This paper specifically seeks to address the topic of controlled biochemical reaction networks. Here a controlled network refers to any model for which the dynamics are influenced by the concentration of a particular reactant that can be considered as an input into the network, within which some given combinations of the reactants can be considered as outputs of the system. In the context of Systems Biology this includes, for example, models of receptor signalling pathways where the concentration of an extracellular ligand may be seen as an input controlling the pathway. The concentration of some subset of the intracellular signalling species may also be considered an output that is directly observed or inferred from some measure of the cellular response. The recently emerging field of Quantitative Systems Pharmacology [45, 46], which proposes to mechanistically model the effects of drug action from the genetic scale upwards is particularly amenable to such a formulation.

Here we develop a model reduction algorithm focused on maintaining the input-output relationship of a controlled biochemical reaction network. The algorithm combines several approaches including conservation analysis, proper lumping and empirical balanced truncation. For controlled systems with a specified output, empirical balanced truncation is designed to give a reduction that accurately maintains the input-output relationship. Unfortunately, due to the need to repeatedly simulate the system under a range of perturbed conditions, empirical balanced truncation can be highly sensitive to model stiffness. Hence, given the stiffness that is commonly associated with such systems, in the combined algorithm proper lumping is employed as a preconditioner to enable the application of empirical balanced truncation whilst retaining an accurate reduced model.

Our paper is structured as follows: we will first outline the general model reduction problem and then proceed to provide an overview of conservation analysis, empirical truncation, and proper lumping. A detailed account of how these methods can be brought together to obtain more accurate reductions than can be obtained via application of any single method in isolation will then be given. Finally, we demonstrate the algorithm via application to two examples: an 11 dimensional model of bacterial chemotactic signalling in *Escherichia coli* [47] and a 99 dimensional model of extracellular signal-regulated kinase (ERK) phosphorylation mediated via the epidermal growth factor (EGF) and nerve growth factor (NGF) receptor pathways [48]. Results are compared and a number of enhancements to the core methods are discussed.

### Problem outline

The models we seek to address are comprised of systems of coupled, nonlinear ODEs. These are formulated as initial value problems and can be expressed by a control affine, state-space representation such that

1a $$\begin{array}{*{20}l} \dot{\boldsymbol{x}}(t) &= \boldsymbol{f}(\boldsymbol{x}(t)) + \sum_{i=1}^{l}\boldsymbol{g}_{i}(\boldsymbol{x}(t)) u_{i}(t),  \end{array} $$

1b $$\begin{array}{*{20}l} \boldsymbol{y} (t) &= \boldsymbol{h}(\boldsymbol{x} (t)),  \end{array} $$

with initial conditions ***x***(0)=***x***_0_ and where the over-dot represents the time derivative, such that $\dot {\boldsymbol {x}}=\frac {\mathrm {d}\boldsymbol {x}}{\mathrm {d}t}$. Here the state variables $\boldsymbol {x}(t)\in \mathbb {R}^{n}$ typically represent the time-varying concentrations of the modelled species, $\boldsymbol {u}(t)\in \mathbb {R}^{l}$ (such that *u*_*i*_(*t*)∈***u***(*t*)) represent the input variables, and $\boldsymbol {y}\in \mathbb {R}^{p}$ represent the output variables. Here ***f***(***x***(*t*)) is the set of functions describing the dynamical interaction between the individual reactants, each set of functions ***g***_*i*_(***x***(*t*)) describes the dynamical behaviour of the reactants interacting with each of the inputs, and ***h***(***x***(*t*)) describes the combinations of the reactant concentrations corresponding to each of the outputs.

We seek a reduced model of the form

2a $$\begin{array}{*{20}l} \dot{\tilde{\boldsymbol{x}}}(t) &=\tilde{\boldsymbol{f}}(\tilde{\boldsymbol{x}}(t))+ \sum_{i=1}^{l} \tilde{\boldsymbol{g}}_{i}(\tilde{\boldsymbol{x}}(t))u_{i}(t),  \end{array} $$

2b $$\begin{array}{*{20}l} \bar{\boldsymbol{y}}(t) &= \tilde{\boldsymbol{h}}(\tilde{\boldsymbol{x}}(t)), \end{array} $$

where $\tilde {\boldsymbol {x}}\in \mathbb {R}^{\tilde {n}}$ represents a reduced set of state variables (such that $\tilde {n}<n$) and for which, given a set of inputs ***u***(*t*), the reduced set of outputs $\bar {\boldsymbol {y}}(t)$ represents an approximation of the original set ***y***(*t*). Similarly to ***f***, ***g***, and ***h*** in the unreduced model, $\tilde {\boldsymbol {f}}(\tilde {\boldsymbol {x}}(t))$ and $\tilde {\boldsymbol {g}}_{i}(\tilde {\boldsymbol {x}}(t))$ are sets of functions describing the dynamical effects of interactions between the reduced state-variables and inputs. Meanwhile $\tilde {\boldsymbol {h}}(\tilde {\boldsymbol {x}}(t))$ approximately maps the reduced state-variables to the outputs.

The accuracy of a reduced model in capturing the dynamics of the original can be defined in a number of ways to suit the needs of the modeller. The most common approaches, however, are based upon the instantaneous error between the outputs of the two systems

3 $$ \boldsymbol{\epsilon}(t)=\left| \boldsymbol{y}(t) - \bar{\boldsymbol{y}}(t)\right|.   $$

The most common metrics are the *L* ^2^-norm $\left \|\boldsymbol {\epsilon }(t)\right \|_{2} = \left (\int \boldsymbol {\epsilon }(t)^{2} \; dt\right)^{1/2}$ or the *∞*-norm ∥***ε***(*t*)∥_*∞*_=sup{***ε***(*t*)}. Throughout this paper we employ a form of maximal relative error *E* as the metric that we aim to minimise, such that

4 $$ \epsilon_{i} \in \boldsymbol{\epsilon}: \:\: \epsilon_{i}(t) = \frac{\left\| y_{i}(t) - \bar{y}_{i}(t)\right\|}{y_{i}(t)} \;\; \text{and} \;\; E = \left\|\boldsymbol{\epsilon}(t)\right\|_{\infty}.   $$

Here, the relative error is selected such reduced models can be compared fairly for a range of different perturbation magnitudes applied both to the inputs and the initial condition of the state-variables.

## Methods

Our combined model reduction algorithm is designed to bring together the methods of nondimensionalisation, conservation analysis, proper lumping and empirical balanced truncation. At its core the method employs proper lumping as a preconditioner (to reduce model stiffness) prior to the application of empirical balanced truncation. In this section we briefly review the variants of the methods employed before providing a more detailed account of the algorithm.

### Nondimensionalisation

Nondimensionalisation refers to the process of rescaling the variables in a system such that the physical units (typically units of concentration and time) are removed from the model [49]. There are number of purposes for nondimensionalisation in the analysis of biochemical systems — primary amongst these is its use in accessing characteristic or intrinsic properties of the reaction network. Usually these are ratios of kinetic rate constants and conserved values that enable greater intuition into how the parameterisation of a model governs its behaviour.

This yields a nondimensionalised parameter set $\tilde {\boldsymbol {p}}$ with entries representing ratios of the original parameters ***p***. Often, nondimensionalisation can result in a reduction in the dimension of the new parameter set $\tilde {\boldsymbol {p}}$ by finding ratios that are fixed to 1 irrespective of the original parameterisation. This does not, however, result in a reduction in the number of modelled reactions or reactants and hence does not reduce complexity as previously defined. Additionally, the dimensionless parameters may lose their innate biological meaning as the ratios they represent may not always hold particular biological significance.

There is usually a large possible number of combinations of the original parameters that could be used to yield these nondimensional ratios - for example, it is common to rescale time relative to a single kinetic rate parameter, amongst which a great number of choices may exist. Whilst there is no single method to determine a ‘best’ or ‘optimal’ nondimensionalisation, in the case where the system is fully parameterised and parameter values are fixed, selecting a nondimensionalised parameter set $\tilde {\boldsymbol {p}}$ with entries all of a similar order will typically improve the numerical properties of the model for computational issues such as rounding error. To achieve this, the combined model reduction algorithm randomly samples 50 possible parameter combinations as nondimensionalsations and selects the one that minimises *σ*, where

5 $$ \sigma = \log_{10}\left(\frac{\text{max}(\tilde{\boldsymbol{p}})}{\text{min}(\tilde{\boldsymbol{p}})}\right).  $$

### Conservation analysis

It is common in models of biochemical reaction networks for the total concentration of certain subsets of the species to remain constant at all times independent of the model’s specific parameterisation [50]. Such subsets are commonly referred to as conserved moieties. Replacement of state-variables via the algebraic exploitation of conservation relations is a common first step in the analysis of biochemical reaction networks. Eliminating one of the conserved state-variables for each of the conservation relations can be used to yield a simplified realisation of the system.

In principle, all such conservation relations for a given biochemical reaction network can be determined by finding the linear dependencies in the associated stoichiometry matrix of the system. Mathematically this relies upon computing the left null space of the stoichiometry matrix for which a number of well-established methods exist. A review of a range of such techniques, including Gaussian elimination and singular value decomposition, can be found in Sauro and Ingalls [51]. For smaller scale systems such methods will usually find all available conservation relations, but for higher dimensional systems difficulties may occur. In particular, it is often necessary to select more stable computational methods to avoid missing conservation relations or finding false ones due to numerical error. A particularly stable method based upon the construction of a QR decomposition via Householder reflections has been developed by Vallbhajosyula et al. [52]. We therefore employ a form of this approach here, a more detailed mathematical treatment of which can be found in Section 1.2 of the Additional file 1.

This form of analysis leads to a simplified realisation of the system under which it is possible to obtain non-singular Jacobians for given states of the model. As such it can be seen as a first step in model reduction schemes involving numerical methods.

### Proper lumping

**Fig. 1:**
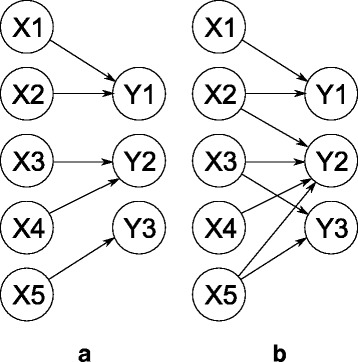
Proper vs improper lumping. **a** Proper lumping: each of the original state-variables (the left column) corresponds to, at most, one of the lumped state-variables (the right column). **b** Improper lumping: each of the original states can correspond to one or more of the lumped state-variables

A lumping can potentially refer to any direct mapping $L: \mathbb {R}^{n} \rightarrow \mathbb {R}^{\tilde {n}}$ of the original state variables $\boldsymbol {x}(t)\in \mathbb {R}^{n}$ to a reduced set $\tilde {\boldsymbol {x}}(t)\in \mathbb {R}^{\tilde {n}}$ where $\tilde {n}<n$. Here we limit ourselves to linear lumping, such that we have a projection of the form

6 $$ \tilde{\boldsymbol{x}}(t) = L\boldsymbol{x}(t),  $$

and, additionally, proper lumping such that the projection *L* becomes a matrix $L\in \left \{0,1\right \}^{\tilde {n}\times n}$ where each column is pairwise orthogonal. This implies that each of the original state-variables corresponds to, at most, one of the lumped state-variables in the reduced model as is depicted in Fig. 1(a).

The dynamics of the reduced variables $\tilde {\boldsymbol {x}}(t)$ are obtained via application of the Galerkin projection [53] (a detailed account of which can be found in the Section 1.1 of the Additional file 1) to the original system (2), such that

7a $$\begin{array}{*{20}l} \dot{\tilde{\boldsymbol{x}}}(t) =&\ L\boldsymbol{f}(\bar{L}\tilde{\boldsymbol{x}}(t)) + \sum_{i=1}^{l} L\boldsymbol{g}_{i}(\bar{L}\tilde{\boldsymbol{x}}(t))u_{i}(t), \\ &\text{with}\ \tilde{\boldsymbol{x}}(0)= \tilde{\boldsymbol{x}}_{0} = L\boldsymbol{x}_{0},  \end{array} $$

7b $$\begin{array}{*{20}l} \tilde{\boldsymbol{y}} (t) =&\ \boldsymbol{h}(\bar{L}\tilde{\boldsymbol{x}}(t)),  \end{array} $$

where $\bar {L}\in \mathbb {R}^{n\times \tilde {n}}$ represents a generalised inverse of *L* such that $L\bar {L}=I_{\tilde {n}}$ (the $\tilde {n} \times \tilde {n}$ identity matrix). An approximation for the original state variables from the reduced variables can therefore be computed as

8 $$ \boldsymbol{x}(t) \approx \bar{L}\tilde{\boldsymbol{x}}(t).  $$

#### Finding an optimal lumping

There exist a number approaches for selecting an appropriate lumping matrix *L* for producing a system of given reduced dimensionality $\tilde {n}$. Here we choose to employ the scheme described by Dokoumetzidis and Aarons [37]. This algorithm runs an exhaustive search of possible lumping matrices to determine which produces the lowest error between simulation of the original model and the reduced model. To speed up this process, it is assumed (from justifications given in the original paper) that the lowest error *k* dimensional reduction obtained via lumping of an *n* dimensional system can also be found as the optimal lumping of two states in the *k*+1 dimensional reduction. This yields a ‘forward selection’ strategy, where 2 of the state-variables are lumped at each step, which greatly decreases the combinatorial burden of possible lumping matrices that must be evaluated.

#### Lumping and stiffness

Stiffness here is defined as the ratio between the largest and smallest eigenvalues of the Jacobian matrix of the system evaluated at its unperturbed initial condition, such that

9 $$ \chi=\frac{\lambda\left(\left.J\right|_{\boldsymbol{x}=\boldsymbol{x}_{0}}\right)_{\text{max}}}{\lambda\left(\left.J\right|_{\boldsymbol{x}=\boldsymbol{x}_{0}}\right)_{\text{min}}}, \:\:\: \boldsymbol{x}_{0} \in \mathbb{R}^{n}.   $$

A high stiffness coefficient implies that parts of the system are evolving at significantly greater rates than others which can lead to traditional methods of simulation being numerically unstable.

The lumping algorithm of Dokoumetzidis and Aarons [37] will tend to sum together those state-variables that interact on faster timescales than their neighbours, and hence rapidly reach a form of proportional equilibrium. As a result the reduced model will tend to contain a lower range of timescales and a lower stiffness coefficient with every additional dimension eliminated. Furthermore, proper lumping, which creates reduced state-variables as straightforward sums of the originals, retains a degree of a biological interpretability that may not hold for alternative, coordinate transforming methods of model reduction.

### Empirical balanced truncation

Our approach to empirical balanced truncation is based upon the procedure developed by Hahn and Edgar [54]. The aim is to construct two covariance matrices, known as the empirical controllability and observability Gramians, via repeated simulations of the system under perturbations of the input and the initial conditions respectively. The controllability Gramian provides information on how changes in the input will alter the state of the system. The observability Gramian provides information on the magnitude of the output any given initial condition of the system can produce. As these Gramians are positive semi-definite matrices, one good way to interpret them is as ellipsoids in the phase-space of the model [55]. The ellipsoid of the controllability Gramian represents the set of states that can be reached for a given magnitude of input, the ellipsoid of the observability Gramian describes the set of initial states that can produce an output of a given magnitude. A detailed mathematical account of the computation of empirical Gramians can be found in Section 1.3 of the Additional file 1.

We have modified the empirical balanced truncation procedure of Hahn and Edgar to deal with a system where state-variables have been eliminated via conservation analysis. In particular the conserved totals should be treated as functions of the initial conditions of the system and be altered in accordance with the perturbations used to create the observability Gramian. This allows us to perturb the initial conditions of the system without risking violation of conservation.

Once the Gramians have been computed the aim is to construct a balancing transformation of the state variables which also acts to equalise and diagonalise the Gramians. As the Gramians are now diagonal, each of the associated state-variables is therefore orthogonal in terms of their contribution to the input-output relationship. Hence, under this transformation, the state-variables which contribute least can be truncated without influencing the remaining terms.

The balancing transformation can be obtained in a numerically stable way following the approach of Laub et al. [56]. Firstly, perform a Cholesky factorisation of both the controllability Gramian $\mathcal {P}$ and the observability Gramian $\mathcal {Q}$ to obtain

$$\mathcal{P} = L^{\intercal}L \:\:\: \text{and} \:\:\: \mathcal{Q} = R^{\intercal}R, $$

where *L* and *R* represent the upper triangular factors of the Gramians. Now, take a singular value decomposition of the newly formed matrix *LR* ^*T*^ and select a reduced dimensionality $\tilde {n}$ of the new model to obtain

$$LR^{\intercal}=\left(U_{1} \; U_{2} \right) \left(\begin{array}{cc} \Sigma_{1} & 0\\ 0 & \Sigma_{2} \end{array}\right) \left(\begin{array}{c} V^{\intercal}_{1}\\ V^{\intercal}_{2} \end{array}\right), $$

where *U*_1_ is an $n\times \tilde {n}$ matrix, *Σ*_1_ is an $\tilde {n}\times \tilde {n}$ matrix (of the form $\Sigma _{1} = \text {diag}(\sigma _{1}^{2},\ldots,\sigma _{\tilde {n}}^{2})$) and $V^{\intercal }_{1}$ is a $\tilde {n}\times n$ matrix. Finally, set

10 $$ T_{1} = \Sigma_{1}^{-\frac{1}{2}}V^{\intercal}_{1}R \:\:\: \text{and} \:\:\: S_{1} = L^{\intercal}U_{1}\Sigma_{1}^{-\frac{1}{2}}.  $$

Given the state-variable projection $\tilde {\boldsymbol {x}}=T_{1}\boldsymbol {x}$ and the particular generalised inverse leading to the approximation $\boldsymbol {x} \approx S_{1} \tilde {\boldsymbol {x}}$, we construct the reduced dynamics for this system again via application of the Galerkin projection to the original system (2), such that

11a $$\begin{array}{*{20}l} &{}\dot{\tilde{\boldsymbol{x}}}(t) = T_{1}\boldsymbol{f}(S_{1}\tilde{\boldsymbol{x}}(t)) + \sum_{i=1}^{l} T_{1}\boldsymbol{g}_{i}(S_{1}\tilde{\boldsymbol{x}}(t))u_{i}(t), \:\:\: T_{1}\boldsymbol{x}(0)=\tilde{\boldsymbol{x}}_{0},  \end{array} $$

11b $$\begin{array}{*{20}l} &{}\tilde{\boldsymbol{y}}(t) = \boldsymbol{h}(S_{1}\tilde{\boldsymbol{x}}(t)).  \end{array} $$

This reduced system will not only feature fewer state-variables, but will also typically be faster to simulate and contain fewer parameters.

#### Stiffness and empirical balanced truncation

The construction of empirical Gramians via repeated simulation of the system under perturbations of input and initial conditions can be sensitive to numerical error. Where balanced truncation is applied using Gramians with a high degree of associated error, blow-up problems can occur for simulations of the reduced system. This accumulation of numerical error is particularly likely to occur for systems with a high-stiffness coefficient (*χ*≫1).

Given the stiffness reducing property of lumping, however, and its retention of some biological meaning it can potentially be treated as a preconditioning step - enabling the application of more numerically sensitive methods (such as empirical balanced truncation) to previously lumped systems.

### The combined model reduction algorithm

**Fig. 2:**
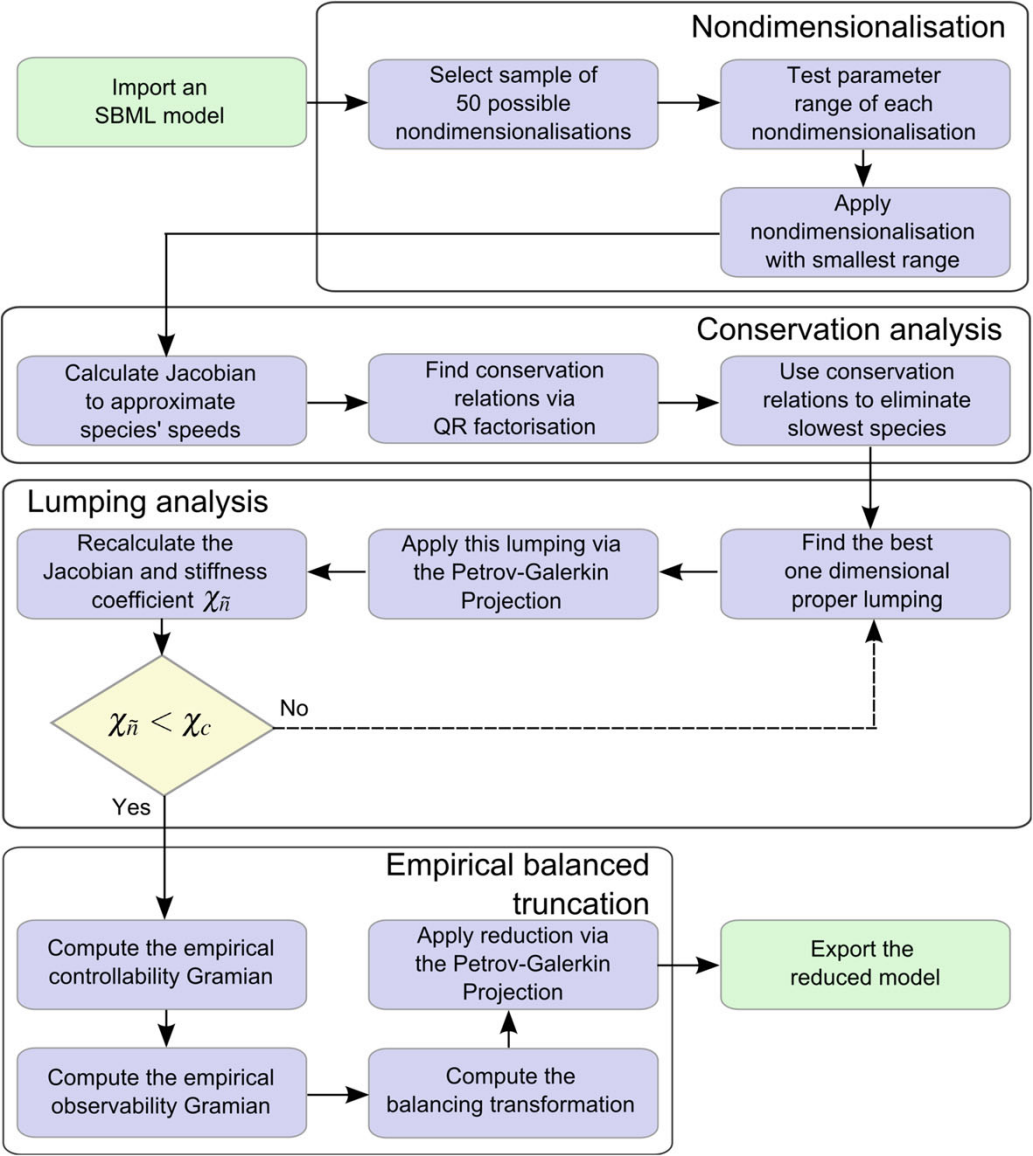
A flow chart giving an overview of the order of steps performed in the combined algorithm. This chart only represents the high-level actions of the algorithm with many of the blocks representing subroutines that are somewhat more nuanced in practice. A detailed account of all steps can be found in the Additional file 1

Here a combined, automatable algorithm for model reduction bringing together the methods of nondimensionalisation, conservation analysis, proper lumping and empirical balanced truncation is introduced. A high-level overview of the algorithm’s steps is shown in Fig. 2.

The algorithm is designed to be automatically applicable to models given in Systems Biology Markup Language (SBML) form — a standardised format for the representation, storage and easy communication of Systems Biology models. Many such models of this form can commonly be obtained from online repositories. Publicly open databases, such as the previously mentioned BioModels Database, contain thousands of such models enabling researchers to share their work in a more accessible way.

As preliminary steps, nondimensionalisation and conservation analysis are applied to the model. Nondimensionalisation is applied to improve numerical accuracy by reducing the range of parameters accounted for in the model. Conservation analysis is then applied in order to obtain a simplified realisation of the system and remove any associated singularities from the system’s associated Jacobian.

At the core of the algorithm, however, are the methods of proper lumping and empirical balanced truncation. Theoretically, empirical balanced truncation should produce lower output error reductions across a range of inputs than lumping. However, due to the need to construct accurate covariance matrices of data from repeated numerical simulations, empirical balanced truncation can fail when applied to highly stiff systems. Conversely, lumping strips the model of some of its stiffness for each reduced dimension. In the combined algorithm, therefore, the complimentary aspects of proper lumping and empirical balanced truncation are exploited. Lumping is used to reduce the system until the stiffness coefficient $\chi _{\tilde {n}}$ of the reduced model is within a satisfactory range $\chi _{\tilde {n}}<\chi _{c}$, for *χ*_*c*_ some pre-chosen critical stiffness value (from numerical experimentation with example models we set this to be 250 in the automated algorithm). Empirical balanced truncation is then employed to obtain further model reduction whilst maintaining a good error bound between the outputs of the original and reduced models.

The algorithm as presented will proceed until the reduced system exceeds the maximum tolerated error, here defined to be 5%. The algorithm then returns the lowest dimensional reduced system that meets this constraint. It is not possible to know a priori what degree of reduction will be attainable by the algorithm, and the actual reduction achieved is both model structure and parameterisation dependent. Application of the combined reduction algorithm does require that the model it is applied to is fully parameterised prior to reduction. As such, the reduced model is only guaranteed to be accurate locally to a point in parameter space. For small perturbations to the initial parameterisation it is likely that the reduced model will remain accurate, however for larger deviations it may not be suitable for describing the overall input-output behaviour of the system.

#### Combined algorithm implementation

The main steps of the combined model reduction algorithm are as follows.

1. Import the model of interest with a set of predefined state-variables ($\boldsymbol {x}(t)\in \mathbb {R}^{n}$), reactions, and rate parameters into the algorithm.
2. Request the user to define the set of inputs (***u***) and the set of outputs (***y***=*h*(***x***(*t*))) in which they are interested.
3. Randomly select a sample of 50 possible nondimensionalisations under the original parameterisation of the model.
4. Calculate the range of the new parameter set for each nondimensionalisation.
5. Select and apply the nondimensionalisation sampled with the smallest range of orders of magnitude of parameters to help avoid rounding-off and truncation errors.
6. Compute the conservation relationships using a standard QR factorization method via Householder reflections (outlined, for example, in [51]).
7. Prioritise which state-variables to replace in the dynamical system via exploitation of the conservation relations.
8. Compute the linear, proper lumping matrix for all lumpable pairs of state-variables remaining in the system. Expressed alternatively, compute the set of all possible proper lumping matrices that will reduce the number of state-variables by one.
9. Compute the lumping inverse.
10. Simulate each lumped system and compute the associated maximal relative output error.
11. Select the lumping matrix *L* and associated inverse that produces the lowest error and apply this reduction.
12. Calculate the stiffness coefficient $\chi _{\tilde {n}}$ for this reduced system.
13. Return to step (8). Exit the loop of steps (8)- (13) when the lumping either violates the maximum tolerated error (typically set to 5*%*) or reduces the stiffness coefficient to within an acceptable range $\chi _{\tilde {n}}<\chi _{c}$ or when the reduced dimensionality $\tilde {n}=1$.
14. Compute the empirical Gramians for the lumped system.
15. Compute the balancing transformation for the given Gramians.
16. Apply the transformation to the model via the Galerkin projection and truncate state-variables until the maximum acceptable error is reached.
17. Produce the symbolic form of the reduced model and terminate.

The main algorithm was implemented in a commercial software package (Matlab 2013b, MathWorks Inc., Natick, MA). To enable the importing and manipulation of models stored in SBML, additional use was made of two open-source toolboxes - libSBML [57] and SBtoolbox2 [58]. Note that a more detailed account of the steps and implementation of the combined algorithm can be found in Section 3 of the Additional file 1.

#### Algorithm enhancements

The algorithm additionally features two specific enhancements to the combined methodologies that will be outlined in the following section. The first of these addresses the question of how to select which state-variables should be explicitly eliminated from the system of ODEs via the exploitation of conservation relations. The second addresses the question of which of the possible generalised inverses should be used to reduce the system via lumping with the Galerkin projection.

##### Selection of state-variables for elimination via conservation analysis:

Typically, the most accurate possible proper lumping of two state-variables in a model will occur between those two that most rapidly equilibrate with respect to the remainder of the system. In short, if two variables quickly reach a point where their proportional concentration with respect to each other is approximately constant, they can be lumped accurately. If a state-variable from this ‘best’ lumped pair has been replaced by an algebraic equation from the conservation relations, then it will necessarily be unavailable for lumping in the combined algorithm. Another concern is that the choice of state-variables eliminated will have an effect upon the reduction of stiffness attainable via the application of lumping; if the fastest interacting state-variables are not represented explicitly the stiffness will require many more steps of lumping in order to be reduced.

To avoid this issue the combined algorithm initially ‘speed’ ranks the state-variables in the model such that the slowest variables can then be selected for elimination. This speed ranking is achieved via numerical calculation of the Jacobian evaluated at the steady-state $\boldsymbol {x}=\boldsymbol {x}^{\ast }_{\boldsymbol {u}_{0}}$ of the system attained under an unperturbed input ***u***=***u***_0_. The absolute value of the diagonal entries of the Jacobian $\left. J\right |_{\boldsymbol {x}=\boldsymbol {x}^{\ast }_{\boldsymbol {u}_{0}}}$ are then used as an approximate metric of the initial time-scale or speed of each of the state-variables. This is equivalent to the outgoing rate of concentration from each state-variable at the unperturbed steady-state of the network. Those state-variables corresponding to the largest values are deemed fast, whilst those corresponding to the smallest values are deemed slow and prioritised for elimination via application of conservation analysis.

##### Alternative lumping inverses:

In order to apply a lumping *L* under the Galerkin projection, some generalised right-inverse $\bar {L}$ of *L* such that $L\bar {L}=I_{\tilde {n}}$ is required. Note that, as $\bar {L}$ could potentially be any generalised right-inverse of *L*, and there exists an infinite number of ways to construct such a matrix. However, the particular choice made will have an effect on the maximal relative output error incurred by the reduced model as defined by Eq. (4). Some of the alternatives are evaluated here.

In the original Wei and Kuo papers [59, 60] they suggest selecting the $\bar {L}$ that reconstructs the steady-state of the system such that $\boldsymbol {x}(t) = \bar {L}\tilde {\boldsymbol {x}}(t)$ for *t*→*∞*. This can be constructed as follows. Let

$$X:=\text{diag}\left\{\boldsymbol{x}^{\ast}\right\}, $$

where ***x*** ^∗^ represents the steady-state of the system such that $ {\lim }_{t \to +\infty } \boldsymbol {x}(t) = \boldsymbol {x}^{\ast }$, then

12 $$ \bar{L} = XL^{\intercal}\left(LXL^{\intercal}\right)^{-1}.   $$

As, in the case of a controlled biochemical network, the steady-state of the system is necessarily dependent on the value of the inputs ***u***(*t*). We here seek to maintain the generality of the reduced model by employing the steady-state attained under an unperturbed input ***u***=***u***_0_, such that $\boldsymbol {x}=\boldsymbol {x}^{\ast }_{\boldsymbol {u}_{0}}$.

The Dokoumetzidis and Aarons [37] paper, which introduces the proper lumping algorithm that the scheme presented here is based upon, follows the work of Li and Rabbitz [61]. They suggested the somewhat simpler approach of using the Moore-Penrose inverse of *L* such that $\bar {L}=L^{+}$, which can similarly be computed as

13 $$ L^{+} = L^{\intercal}\left(LL^{\intercal}\right)^{-1}.   $$

Typically the approach of reconstructing the steady-state values will lead to a better approximation of the original dynamics. In the case where a lumping sums a group of species all with a steady-state value of zero, however, this will lead to a singularity and thus the Moore-Penrose approach is to be preferred.

We thus consider an alternative to the above methodologies; we design an inverse $\hat {L}$ designed to reconstruct the proportionally average value of the states-variables for all time-points with non-zero values. To understand this alternative lumping inverse, first observe that all lumping matrices *L* (and inverses $\bar {L}$) can be expressed as the composition of sequential lumping matrices of two state-variables. For example

$$\left[ \begin{array}{cccc} 1&1&1&0\\ 0&0&0&1 \end{array}\right] = \left[ \begin{array}{ccc} 1&1&0\\ 0&0&1 \end{array}\right] \left[\begin{array}{cccc} 1&1&0&0\\ 0&0&1&0\\ 0&0&0&1 \end{array}\right], $$

which demonstrates that a lumping of 3 state-variables can be achieved via two sequential lumpings of two state-variables. The novel inverse $\hat {L}$ is constructed for the two state-variable lumping case and can be generalised to any lumping via this process of sequential composition. Hence consider the general case seeking to lump the state-variables *x*_*h*_(*t*) and *x*_*k*_(*t*) (with *h*<*k*), where the corresponding lumping matrix *L*={*l*_*ij*_} has the entries

14 $$ l_{ij} = \left\{\begin{array} {ll} 1 & \text{for}~i=j~\text{with}~i<k,\\ 1 & \text{for}~i=j-1 ~\text{with}~ i\geq k, \\ 1 & \text{for}~i=h,j=k,\\ 0 & \text{otherwise.} \end{array}\right.  $$

The lumping inverse $\hat {L}= \left \{\hat {l}_{ij}\right \}$ is then based upon calculating the average proportion of each of the lumped state-variables *x*_*h*_(*t*) and *x*_*k*_(*t*) during the interval 0≤*t*≤*T*; here, *T* is defined as some time point where the system can be assumed to have approximately reached steady-state, such that

$$\left\| \boldsymbol{x}(T) - \boldsymbol{x}^{\ast}\right\|\ll 1. $$

Hence each element of the inverse is constructed as follows

15 $$ {}\hat{l}_{ij} = \left\{\begin{array}{ll} 1 & \text{for}~i=j ~\text{with}~ i<h,\\ 1 & \text{for}~i=j ~\text{with}~ i>h~\text{and}~j<k,\\ 1 & \text{for}~i=j+1 ~\text{with}~ i\geq k, \\ \frac{1}{T}\int_{0}^{T}\frac{x_{i}(t)}{x_{i}(t)+x_{j}(t)}dt &~\text{for}~i=h,j=h,\\ \frac{1}{T}\int_{0}^{T}\frac{x_{j}(t)}{x_{i}(t)+x_{j}(t)}dt &~\text{for}~i=k,j=h,\\ 0 & \text{otherwise.} \end{array}\right.  $$

In the case where the time *T*→*∞* and either or both state-variables have a non-zero steady-state this reduces to the steady-state reconstructing lumping inverse defined by Eq. (12). For the zero steady-state situation, however, it avoids the issue of numerical singularities.

#### Indices of controllability and observability

One of the benefits of the application of empirical balanced truncation is its potential use in obtaining metrics of observability and controllability for the individual state-variables of nonlinear systems. Given the typically nonlinear nature of cell signalling models, this potentially allows a framework for accessing indices of the controllability of each state-variable via receptor activation or suspension, the observability of each state-variable in influencing the output of interest, and the contribution of each state-variable to the overall input-output relationship of the model.

The interpretation of standard Gramians as ellipsoids describing the controllability and observability of the directions in phase-space [55] can also be extended to the interpretation of empirical Gramians. Hence it is possible to employ these matrices to obtain indices of controllability and observability for individual state-variables in nonlinear systems. Given this, we define the following indices.

##### Observability index:

The observability index of the *i*-th state-variable *ν*_*oi*_∈***ν***_*o*_ is defined as

$$\nu_{oi} := \frac{\sqrt{\boldsymbol{e}^{\intercal}_{i}\mathcal{Q}\boldsymbol{e}_{i}}}{ \text{max}\left\{\sqrt{\boldsymbol{e}^{\intercal}_{i}\mathcal{Q}\boldsymbol{e}_{i}}\right\}}, $$

where 0≤*ν*_*oi*_≤1 and ***e***_*i*_ represents the *i*th unit vector. Note the index has been rescaled relative to the maximally observable state-variable, such that each represents the comparable influence of the associated state-variable. The greater the value the more influence the state-variable has on the observed output. A value of zero indicates the state-variable has no effect on the output of interest.

##### Controllability index:

The controllability index of the *i*-th state-variable *ν*_*ci*_∈***ν***_*c*_ is defined as

$$\nu_{ci} := \frac{\sqrt{\boldsymbol{e}^{\intercal}_{i}\mathcal{P}\boldsymbol{e}_{i}}}{\text{max}\left\{\sqrt{\boldsymbol{e}^{\intercal}_{i}\mathcal{P}\boldsymbol{e}_{i}}\right\}}, $$

with 0≤*ν*_*ci*_≤1. Again, the indices here are rescaled relative to their maximal value such that they are more comparable to each other and to other indexes. The greater the value the more controllable the state-variable is via the input. A value of zero indicates that the input has no effect on the corresponding state-variable.

##### Input-output importance index:

The input-output index ***ν*** is then defined as the element-wise product of the observability and controllability indices rescaled proportionate to the maximal value. Hence the index corresponding to the *i*-th state-variable *ν*_*i*_∈***ν*** is given by

$$ \nu_{i} := \frac{\nu_{oi}\nu_{ci}}{\text{max}\left\{\nu_{oi}\nu_{ci}\right\}}, $$

with 0≤*ν*_*i*_≤1. This therefore provides an overall metric of the corresponding state-variable’s influence on the overall input-output relationship of the system.

## Results and discussion

In this section two examples are employed to demonstrate the application of the combined model reduction algorithm, the enhancements made to the base methods, and the calculation of the indices of controllability and observability. The first of these systems is a relatively simple (11 dimensional) model of bacterial chemotaxis in *Escherichia coli* - the modest scope of this model allows the application of our methods to be more easily intuited. The second is a significantly more substantial model (99 dimensional) describing the mediation of ERK activation via both the EGF and NGF receptor pathways. Through application to a model of this size, our methods demonstrate their potential usefulness in analysing models that might be inscrutable under traditional approaches.

### A model of bacterial chemotaxis

We have applied our combined model reduction methodology to a model of chemotactic signalling in *E. coli* as detailed in Tindall et al. [47] and summarised in Section 2 of the Additional file 1. This is a modest 11 dimensional model detailing a system of 12 biochemical reactions. This serves as a reasonable starting example as the model is intuitively tractable, but also of a sufficient size and complexity to be meaningfully reduced by the combined algorithm.

*E. coli* is a common, rod shaped, gram-negative bacterium often used as a model organism in biological studies due to both the large body of existing literature characterising its behaviour as well as the relative ease and inexpensiveness in its growth and experimental manipulation. There are many strains of *E. coli* present in nature, but the model discussed here pertains specifically to those strains that exhibit a chemotactic response. Chemotaxis is the process by which a cell senses an environmental chemical gradient and biases its movement towards those regions most suitable for growth and reproduction. In the model presented here, this process involves the transmembrane receptors on the surface of the bacterium sensing the local concentrations of an attractant or repellent; a decrease in attractant or an increase in repellent will cause the receptors to activate a signalling pathway inside the cell resulting in an increase of the intracellular concentration of the phosphorylated chemotactic CheY protein, referred to here as CheY_*P*_. This concentration, in turn, modulates the flagellum’s movement, resulting in a change of direction for the cell either towards attractants or away from repellents.

Chemotaxis represents a good example model to work with as its attractant-receptor behaviour represents a controlled system, and it is highly amenable to the input-output problem formulation that the combined algorithm seeks to address. For our analysis, the external concentration of some chemotactic attractant was treated as the input into the system and the total concentration of CheY_*P*_ (in both free and complex forms). Hence,

16 $$ y(t) = [\text{CheY}_{P}] + [\text{CheA}\cdot \text{CheY}_{P}] + [\text{CheY}_{P}\cdot \text{CheZ}].  $$

The model has a stiffness coefficient at the initial condition of the system of 958.3, which is relatively high. Values for these initial conditions can be found in Section 2 of the Additional file 1.

#### Reduction

We sought to compare the reduction of this example via lumping and empirical balanced truncation alone, and our combined algorithm.

In respect of the combined algorithm, the process of reduction began by nondimensionalising the variables of the system - specifically seeking to rescale the initial conditions and coefficients in the model such that they spanned the fewest orders of magnitude with the aim of avoiding possible issues of numerical truncation. The conservation relations for the system were then calculated; as 4 conservation relations were found, this lead to a system of 7 ODEs that exactly replicated the original system’s dynamics.

The algorithm then lumped the state-variables until the stiffness coefficient was less than 250. In our example this occurred at 5 state-variables. Finally, empirical balanced truncation was applied to the 5 dimensional reduced model. In this case, the empirical Gramians were constructed using data from 100 distinct simulations covering perturbations to both the models input parameter *u*(*t*) and the initial conditions ***x***_0_. In both cases, perturbations were uniformly sampled from 0.2 to 1.8 times each parameter’s original, unperturbed value. Using the balancing transformation and straightforward truncation the associated error for each possible dimensionality of reduction (between four and one) was calculated. A significantly more detailed account of reducing the bacterial chemotaxis model can be found in Section 2.1 of the Additional file 1.

**Fig. 3:**
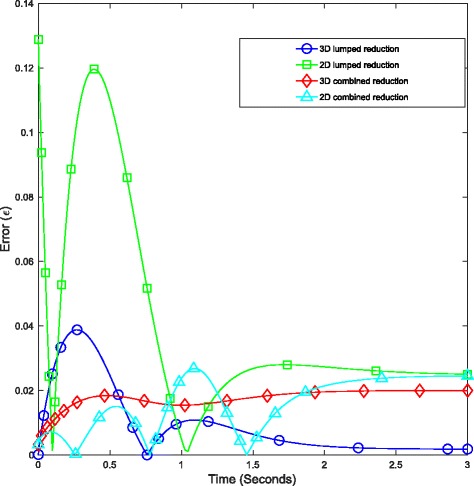
Relative error between the original and reduced models of *E. coli* chemotaxis. These systems were simulated to steady-state under the introduction of 10*μ* *M* concentration of extracellular attractant at *t*=0. Figure depicts time varying errors incurred for the 3 and 2 dimensional reduced models under lumping applied in isolation and via the combined reduction algorithm

**Fig. 4:**
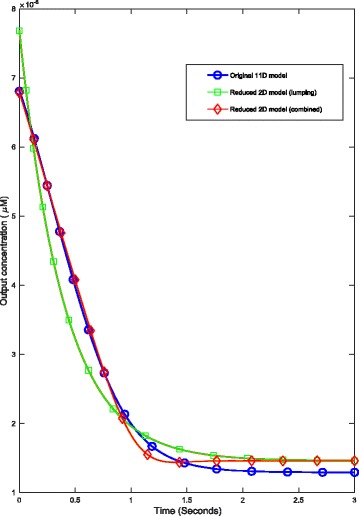
Timecourse of output from the original 11-dimensional and reduced, 2-dimensional models of *E. coli* chemotaxis. Here, the systems were simulated to steady-state under the introduction of 10*μ* *M* concentration of extracellular attractant at *t*=0

The results in Table 1 along with Figs. 3 and 4 clearly demonstrate the combined algorithm produces more accurate reductions than those of either method in isolation. At the 2-dimensional reduction level, the combined algorithm exhibited an approximately 78*%* improvement in reduction error in comparison to the use of lumping alone. Additionally, this reduced model produced a significant speed-up in simulation time. In the case of 100 repeated simulations over a 3 second period under the introduction of 10*μ* *M* concentration of attractant ligand at *t*=0, the original 11 dimensional system required 0.2594 seconds on average to be simulated, whilst the 2 dimensional reduced model was solved in 0.0101 seconds.

**Table 1:** Error results for the nonlinear methods of model reduction applied to the *E. coli* chemotaxis model

Number of	Empirical balanced	Lumping	Combined method
dimensions	truncation		
6	1.396*%*	0.15*%*	-
5	1.655*%*	0.51*%*	-
4	*#*	0.54*%*	0.51*%*
3	*#*	4.77*%*	2.00*%*
2	*#*	12.88*%*	2.84*%*
1	*#*	75.56*%*	21.86*%*

Note: ‘ *#*’ implies Matlab could not numerically simulate this reduction using ode15s due to stiffness. ‘-’ implies the reduction error at this point was equal to the lumping error. The errors stated represent the maximal relative error between the original and reduced systems when simulated to steady-state under the introduction of 10*μ* *M* concentration of attractant ligand at *t*=0

#### Selection of species eliminated via conservation analysis

As was previously discussed, the selection of species eliminated via conservation analysis can have a substantial effect on model reduction error. The combined algorithm hence applies a speed-ranking step designed to estimate which of the biochemical reactants are likely to be most readily lumped.

To demonstrate the use of such an approach we have calculated the associated error and stiffness coefficients incurred under lumping for the chemotaxis model where the species eliminated via conservation were selected either naively or via the speed-ranking approach. These results can be found in Table 2. In the naive case the algorithm selected species CheA, CheA_*P*_·CheY, CheZ and CheB to be eliminated via conservation. Whilst in the selective case the algorithm selected species CheA, CheA·CheY, CheZ, and CheB_*P*_. These results demonstrate that carefully considering which state-variables should be replaced via conservation relations can greatly improve the overall error incurred via lumping and yield a better reduction in model stiffness.

**Table 2:** Comparison of reduction error and stiffness coefficients at each level of dimensional reduction for the *E coli* signalling model under different approaches to conservation analysis using the combined algorithm

Dimensions	Naive elimination		Selective elimination	
	Lumping error (*ε*)	Stiffness	Lumping error (*ε*)	Stiffness
6	1.37*%*	876.73	0.15*%*	794.91
5	2.45*%*	904.80	0.51*%*	56.22
4	4.8*%*	907.59	0.54*%*	60.16
3	74.18*%*	542.24	4.77*%*	58.95
2	339.99*%*	99.32	12.88*%*	25.95
1	424.33*%*	1	75.56*%*	1

This table compares the effect of either naively or rigorously selecting which species to eliminate via the application of conservation relations. The errors stated represent the maximal relative error between the original and reduced systems when simulated to steady-state under the introduction of 10*μ* *M* concentration of attractant ligand at *t*=0

#### Analysis of alternative lumping inverses

The second enhancement to our combined method concerns the choice of lumping inverse used during model reduction. This topic does not seem to have been well explored in the literature, but as will be demonstrated here this choice can have a sizeable effect on the overall accuracy of a reduced model. In the case of the bacterial chemotaxis model, results comparing the three approaches for selecting a matrix inverse can be found in Table 3. In the case of the average inverse $\hat {L}$ we have set *T*=5 seconds.

**Table 3:** Comparison of maximal relative error under differing lumping inverses for the reduction of the *E coli* model via lumping in isolation

Dimensions	Moore-Penrose	Steady-state	Average
6	6.67*%*	0.15*%*	0.18*%*
5	10.58*%*	0.55*%*	0.51*%*
4	13.99*%*	0.54*%*	1.26*%*
3	26.93*%*	4.78*%*	4.77*%*
2	78.36*%*	13.45*%*	12.88*%*
1	70.92*%*	75.56*%*	82.34*%*

Each row represents a further level of dimensional reduction for the model, whilst the columns represent the different methods of lumping inverse. The ‘Moore-Penrose’ column contains values where the lumping inverse $\bar {L}$ is the Moore-Penrose or pseudoinverse of the lumping matrix *L*. The ‘steady-state’ column contains values where the lumping inverse $\bar {L}$ is selected to reconstruct the unperturbed steady-state values of the original system such that $\boldsymbol {x}^{\ast }_{\boldsymbol {u}_{0}}=\bar {L}\tilde {\boldsymbol {x}}^{\ast }_{\boldsymbol {u}_{0}}$. The errors stated represent the maximal relative error between the original and reduced systems when simulated to steady-state under the introduction of 10*μ* *M* concentration of attractant ligand at *t*=0

These results show that, whilst the Moore-Penrose inverse performs worse than the others, both the steady-state and averaged inverses can produce very good results. Given that the optimal approach can vary between lumping steps, the combined algorithm has been designed in such a way as to trial both the average and steady-state inverse at each step before selecting the most accurate. The overall inverse is then returned as the composition of the sequentially best inverses for each dimensionality of lumping.

#### Indices of controllability and observability

In the 5 dimensional lumped model created via the algorithm, only one lumped state-variable was created. This variable represents the sum of species Y_*P*_, A·Y_*P*_, and A_*P*_·Y_*P*_ which can be thought of as representing the phosphotransfer chain between species i.e. A and Y.

Calculating empirical Gramians for this lumped system yields the indices given in Table 4 (note that indices here can only be explicitly calculated for the retained species that were not eliminated via exploitation of conservation relations). Here the lumped variable, Y_*P*_+A·Y_*P*_+A_*P*_·Y_*P*_, is shown to be the most easily controlled, the most easily observed, and in total the most responsible for carrying the input-output signal from change in attractant concentration through to the phosphorylation of species Y and hence chemotaxis. Additionally, this set of species is readily lumpable, having such a low associated error cost and resulting in a large reduction in model stiffness. This suggests that the phosphotransfer process occurs significantly faster than the remainder of the network and hence equilibrates quickly. This finding concurs with the known biology, and demonstrates that Y_*P*_+A·Y_*P*_+A_*P*_·Y_*P*_ is more significant in the process of chemotactic signalling than the other species described in this model. Also noteworthy is the importance of species A·Y which agrees with other work (i.e. Tindall et al. [47]). Finally, the extremely low observability and controllability of CheB suggests that the overall concentration of it has an almost negligible response to the change of extracellular attractant and very little effect on the output in comparison to the remainder of the network. This also makes sense biologically, as CheB is functionally involved in the process of adaptation (how the cell steadily adjusts to the concentration of the chemotactic attractant), as opposed to being directly involved in the actual chemotactic flagellar response.

**Table 4:** Controllability and observability index values for the model of chemotactic signalling in *E. Coli*

Species	Controllability	Observability	Input-output
CheA·CheY	0.865	0.881	0.762
CheA_*P*_	0.846	0.497	0.421
CheY_*P*_+CheA·CheY_*P*_+CheA_*P*_·CheY	1	1	1
CheY_*P*_·CheZ	0.343	0.703	0.241
CheB_*P*_	0.004	0.006	2×10^−5^

### A model of extracellular regulatory kinase activation

The second example provided here is a model of ERK phosphorylation mediated via the EGF and NGF receptor pathways that was originally detailed in Sasagawa et al. [48]. This biological system commonly arises in the study of cancer and pain, and remains an area of ongoing clinical significance. The SBML representation employed here of this model including the parameterisation and initial conditions is available at www.ebi.ac.uk/biomodels-main/BIOMD0000000049.

This is a relatively large biochemical model describing 150 reactions and 99 species. The model additionally integrates two receptor pathways (EGFR and NGFR) allowing exploration as to how they interact. Due to its size and clinical relevance, this model therefore represents a prime candidate for the application of model reduction techniques. Although fully parameterised systems of this scope remain somewhat uncommon, their occurrence in the literature is increasing; primarily a result of increases in data and knowledge of cellular systems at finer spatial scales. However, even with such data available, approximations may still be required to model parameters particularly in cases where the model acts as a representation of more complex underlying biochemical mechanisms. We have thus employed this system as an example to demonstrate that our methodology remains valid for systems of varying complexity and size, assuming all parameter values are known.

**Fig. 5:**
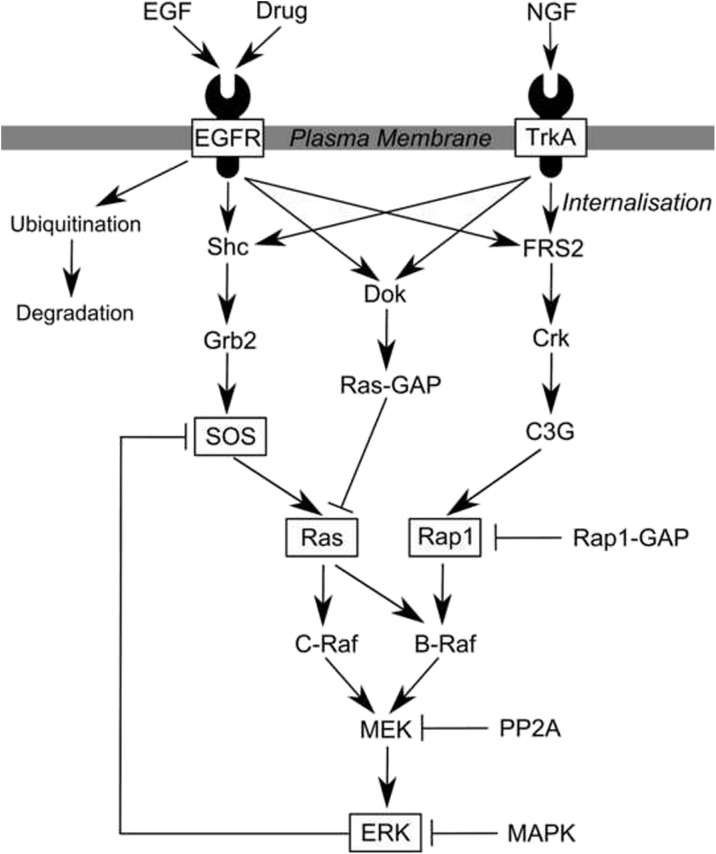
Reproduction of the block schematic depiction of the ERK activation model due to Sasagawa et al. [48]

A full description of the model and its parameterisation can be found in Sasagawa et al. [48]. A block schematic diagram of the system is given in Fig. 5; the blocks in this diagram represent various ‘submodules’ of the system each containing a number of reactants and the reactions that link them. We made one minor modification to the original model; the state-variable representing total concentration of proteasome was altered to have a rate of depletion such that the model was asymptotically stable. Without this proteasome would accumulate indefinitely and cause the system to be unstable.

The analysis performed here treats only one of the pathways as salient, such that the rate of EGF binding represents the only input under consideration. Additionally, the total concentration of phosphorylated ERK (in complex with other species or in isolation) is regarded as the output, such that

$$\begin{aligned} {}y\! = [\!\text{ppERK}] \!+ [\!\text{dppERK}] \!+ [\!\mathrm{ppERK.MKP3}] + [\!\mathrm{dppERK.MKP3}]. \end{aligned} $$

Additionally, the system employs initial conditions such that it begins at a non-zero steady-state with no input into the model (i.e. at the natural rate of EGF binding). Note that we chose to look at the EGF receptor - ERK activation pathway in particular as its dynamical behaviour exhibits an adaptive response [48]. The retention of this nonlinear behaviour in a reduced model serves as a good demonstration of the combined algorithm’s strengths and particular applicability in the field.

**Fig. 6:**
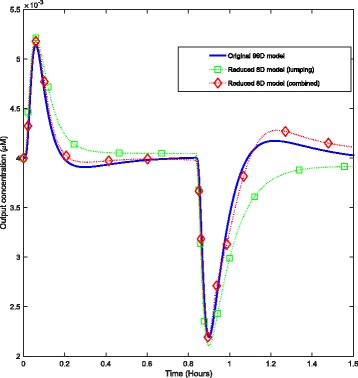
Timecourses of the output from the original 99-dimensional and the reduced 8-dimensional systems. This plot emphasises the fact that the reduced model is designed to remain valid for any reasonable change in input. The system starts by being affected by an agonist that increases the rate of EGF binding by 25*%* for 50 minutes, at this point the input flips to an antagonist decreasing the rate of EGF binding by 50*%* and runs for the same time period. At any given time point the error between the original and reduced model exceeds no more than 5*%*

Conservation analysis was performed via QR factorisation and from which 23 states could be eliminated via the speed-ranking method. Exploitation of these conservation relations resulted in a 76 dimensional model which was then further reduced via the combined algorithm, results of which can be seen in Table 5 and Fig. 6.

**Table 5:** Error results comparing the application of empirical balanced truncation, lumping, and the combined method of model reduction to the ERK activation model

Dimension	EBT error	Lumping error	Stiffness	Combined error
75	0.76*%*	≈0*%* ^∗^	42658	−
50	*#*	0.01*%*	42633	−
25	*#*	0.52*%*	10664	−
15	*#*	1.26*%*	7934	−
14	*#*	2.21*%*	7934	−
13	*#*	2.29*%*	7934	−
12	*#*	1.21*%*	1591	−
11	*#*	3.07*%*	236	−
10	*#*	6.02*%*	264	2.84*%*
9	*#*	10.96*%*	211	4.02*%*
8	*#*	13.12*%*	43	4.32*%*
7	*#*	14.18*%*	42	4.77*%*
6	*#*	29.53*%*	44	13.08*%*
5	*#*	39.03*%*	45	20.81*%*
4	*#*	46.47*%*	212	31.09*%*
3	*#*	54.67*%*	42	34.58*%*
2	*#*	53.52*%*	18	41.10*%*
1	*#*	55.73*%*	1	50.46*%*

Note: ‘ *#*’ implies Matlab could not numerically simulate this reduction using ode15s due to issues associated with stiffness. ‘-’ implies the reduction error at this point was equal to the lumping error. The errors stated represent the maximal relative error between the original and reduced systems when simulated to steady-state under the introduction of agonist increasing the rate of EGF receptor binding by 50*%* at *t*=0. ^∗^ implies that the error was within the numerical tolerance of the simulator

The original model had an extremely high stiffness coefficient at the initial condition of the system of 42658. Via lumping this could be reduced to 235.7 for the 11 dimensional lumped reduction enabling application of empirical balanced truncation. In this case, the empirical Gramians were constructed using data from 50 distinct simulations covering perturbations to both the models input parameter *u*(*t*) and the initial conditions ***x***_0_. In both cases, perturbations were uniformly sampled from 0.4 to 1.6 times each parameter’s original, unperturbed value.

The results shown in Table 5 highlight the extent of the reductions that can be obtained via the combined method with very little associated error. In particular the combined method has again demonstrated that it can produce better reductions than either method in isolation. The 7 dimensional reduced model had only a 4.77*%* associated maximal relative error as compared to the original model when simulated from steady-state under a 50*%* inhibition of EGF receptor binding. This is equivalent to an approximately 68*%* improvement in error over the 7 dimensional reduction achieved by lumping in isolation. Repeated simulation revealed that the original model had an average simulation time of 1.357 seconds, whilst the reduced 7 dimensional model required only 0.144 seconds.

Note that Matlab files regarding the reduction of the ERK activation model can be found online [62].

#### Indices of controllability and observability

Computing the controllability and observability indices for the 11-dimensional lumped version of the ERK activation model yields the results given in Table 6. In comparison to the results for the *E. coli* chemotactic signalling model, the results here are significantly more difficult to intuit. This is primarily due to the fact that the lumped variables often include species from highly disconnected areas of the original network, making it difficult to interpret their biological significance.

**Table 6:** Controllability and observability index values for the model of ERK activation controlled via the EGFR pathway

Lumped	Controllability	Observability	Input-output
state-variable	index	index	index
*x*_1_(*t*)	0.3022	0.0176	0.0053
*x*_2_(*t*)	0.0201	0.0153	0.0003
*x*_3_(*t*)	0.0189	0.0009	1.681×10^−5^
*x*_4_(*t*)	0.8547	0.2752	0.2352
*x*_5_(*t*)	0.6194	0.0079	0.0049
*x*_6_(*t*)	1	1	1
*x*_7_(*t*)	0.1412	0.1043	0.0147
*x*_8_(*t*)	0.1378	0.0018	0.0003
*x*_9_(*t*)	0.1539	0.0164	0.0025
*x*_10_(*t*)	0.0473	0.0092	0.0004
*x*_11_(*t*)	0.3312	0.0442	0.0146

Despite this, however, it is still possible to obtain some insight from the indices calculated. The most controllable and observable lumped state-variable, *x*_6_(*t*), for example, primarily contains the concentration of singly phosphorylated mitogen/extracellular signal-regulated kinase (MEK), in isolation and in its various complex forms. This concurs with the known biology as phosphorylated MEK represents the point in the pathway that all possible routes of activation are directed towards before the phosphorylation of ERK occurs. The second most important state-variable, *x*_4_(*t*), is somewhat more difficult to parse, but is most distinguishable from the other state-variables by its inclusion of the SOS protein and associated complexes and species along this pathway. This, again, concurs with the known biology as SOS represents the most responsive branch of the pathway to EGFR binding that is described by the model. Similarly, the most unimportant state-variable, *x*_3_(*t*), can be seen as disproportionately representing the FRS2-C3G branch of the pathway, which is significantly more responsive to binding of the NGF receptor than that of EGF.

Overall the indices do seem to automatically provide a degree of model intuition that is often not possible for such large systems. As such they represent one possible solution to a core issue in the study of complex models. Due to the nature of the lumping algorithm, however, they are somewhat limited in their usefulness. An alternative approach, which we would hope to explore in future work, would be to constrain the lumping algorithm so as to obtain more readily interpreted lumped variables prior to the calculation of indices.

## Conclusions

In this paper a combined model reduction algorithm incorporating conservation analysis, proper lumping and empirical balanced truncation for the reduction of high dimensional systems of nonlinear ODEs has been presented. This algorithm was designed for specific application to controlled models of biochemical systems. Such models typically have a number of associated properties, including nonlinearity, stiffness and high dimensionality, which any model reduction approach must address. Under the combined algorithm conservation analysis is first used to obtain a simplified realisation of the system, proper lumping is then used to reduce the model whilst also reducing the stiffness coefficient and finally empirical balanced truncation is employed to achieve a more accurate and lower dimensional reduction than could be obtained by further lumping. The algorithm has additionally been designed to be highly automatable; it is implemented so as to take models stored in the SBML format and return highly accurate reduced systems without significant user input.

Whilst the algorithm has broad applicability, crucially, it focuses on the reduction of controlled biochemical systems that are amenable to an input-output formulation. In reducing a model the algorithm seeks primarily to maintain the input-output response profile, such that the reduced model can accurately predict the output of the original model for a wide range of possible inputs. Such an approach may be particularly applicable to the newly emerging field of Quantitative Systems Pharmacology, which aims to mechanistically describe the effects of drug administration across multiple scales of action. Here the preservation of dose-response behaviour is of primary importance and tallies well with the algorithm’s emphasis on maintaining a model’s input-output relationship.

One of the difficulties with reduced models obtained under this approach is that the meaning of the dynamical equations is necessarily obfuscated by the coordinate transformations that have been applied. The combined algorithm starts with a fully mechanistic ‘white box’ model of a signalling pathway, whereby all of the biological reactions and reactants have a one-to-one correspondence with terms in the system of ODEs. The algorithm then produces a reduced ‘grey-box’ model where this biological interpretation of the governing equations is now hidden by the transformations applied. Computationally, however, the reduced model remains an accurate approximation to the original. The reduced state-variables can still be mapped back to the original biological species, thus allowing mechanistic insight to be gained as opposed to solely empirical observation. Hence, whilst the reduced system may no longer serve as a directly interpretable description of the original biological system, it still retains a high degree of utility for the analysis of the model’s overall input-output relationship and its mechanistic underpinnings.

The algorithm in the form presented here assumes that the model in question is initially fully parameterised. It is however possible to extend the algorithm such that the reduced models constructed are guaranteed to remain valid across a range of parameterisations by additionally evaluating perturbations to the parameter values whilst constructing the lumping matrices and the empirical Gramians. Hence, at each step the possible reduced models are tested not only for perturbations to the original input and the initial conditions, but also for perturbations to the parameterisation of the system. To maintain validity across a defined range of values in parameter space, sampling approaches such as latin hypercube sampling would likely work well, but such ideas are not explored in this initial study.

The algorithm was demonstrated via application to two systems; an 11 dimensional model of bacterial chemotactic signalling in *E. coli* and a 99 dimensional model of ERK activation mediated via the EGF and NGF receptor pathways. The results for both models highlight the extent of reduction that can be obtained with very little associated error and therefore the potential usefulness of these model reduction methods in the analysis of Systems Biology models.

From the results it can be seen that both models began with a high stiffness coefficient. As a result, the application of empirical balanced truncation to the unreduced systems yielded higher error than lumping alone and numerically failed after truncating only a small proportion of the state-variables. Crucially, the proper lumping scheme was able to act as a good pre-conditioner and efficiently stripped stiffness from the systems, hence closing eigengaps in the associated Jacobians. Via subsequent application of empirical balanced truncation the combined algorithm was able to produce significantly better reductions than could be obtained by any of the individual methods in isolation. Furthermore, these reduced systems exhibited a significant speed-up in simulation time. In the case of the *E. coli* model we observed a 96.1*%* speed-up in simulation time whilst only incurring a 2.8*%* error. The ERK activation model demonstrated a 89.4*%* speed-up in simulation time in exchange for a 4.77*%* maximal relative error. For applications such as parameter optimisation or agent based modelling approaches, where an extremely large number of simulations may be required, speed-up is a substantial benefit. This speed-up can be attributed to two main factors - the reduction in the number of state variables present in each system and the reduction in their overall stiffness coefficients.

There are still a number of open questions with regards to this work, in particular to what extent does a model reduction (specifically, under a coordinate transformation of the state variables) remain accurate if the parameters in the original system are altered? Additionally, are there reduced models that remain valid over a larger range of parameterisations than others? If now, instead of considering the rate parameters to represent a set of fixed values, we consider the parameters to represent a distribution of physically acceptable values how does this affect our selection of the best reduction? Understanding these questions is a necessary next step in further growing the practical applicability of model reduction for biochemical systems.

The paper also introduced the concept of empirical indices of controllability and observability for biochemical systems; as was demonstrated these indices can be used to provide substantial insight into models of even a very large size. This usefulness, however, is dependent upon the biological interpretation of the specific lumping used to precondition the system. Developing a lumping algorithm specifically for the calculation of these indices represents a reasonable extension of this work.

The combined model reduction algorithm has demonstrated good results for the reduction of controlled, nonlinear, stiff, high-dimensional models of biochemical reaction networks. Reduced systems produced under this approach maintain a high degree of input-output accuracy and see a significant speed-up in simulation time. These results justify research into the combining of complementary reduction methodologies and highlight the use of empirical balanced truncation, which had not previously seen application in the reduction of biochemical systems.

## Additional file

Additional file 1 Provides a mathematical account of the underlying methodologies employed and gives a detailed, stepped proceedure for the application of the combined reduction algorithm. (PDF 631 kb)

## Abbreviations

EGF: Epidermal growth factor
ERK: Extracellular regulatory kinase
MEK: Mitogen/extracellular signal-regulated kinase
NGF: Nerve growth factor
ODE: Ordinary differential equations
SBML: Systems Biology Markup Language
SVD: Singular value decomposition
