# Supplementary material for: A combined model reduction algorithm for controlled biochemical systems
Source: BMC Syst Biol. 2017 Feb 13;11:17. doi: 10.1186/s12918-017-0397-1 (PMC5307760; doi:10.1186/s12918-017-0397-1)
Supplement: Additional file 1 — Provides a mathematical account of the underlying methodologies employed and gives a detailed, stepped proceedure for the application of the combined reduction algorithm. (PDF 631 kb) [file 12918_2017_397_MOESM1_ESM.pdf]

A combined model reduction algorithm for controlled biochemical systems – supplementary information

Thomas J. Snowden[1], Piet H. van der Graaf[2], Marcus J. Tindall[1,3,*]
1 Department of Mathematics and Statistics, University of Reading, Reading, UK
2 Leiden Academic Centre for Drug Research, Universiteit Leiden, Leiden, Netherlands
4 The Institute for Cardiovascular and Metabolic Research (ICMR), University of Reading, Reading, UK
$*$ **E-mail: M.Tindall@reading.ac.uk**

Contents

1 Overview of model reduction methods **1**
1.1 The Galerkin projection . . . 1
1.2 Conservation analysis . . . 2
1.3 Empirical balanced truncation . . . 4

2 Chemotaxis model summary **5**
2.1 Reduction . . . 8
2.1.1 Nondimensionalisaton . . . 8
2.1.2 Conservation Analysis . . . 8
2.1.3 Lumping . . . 10
2.1.4 Empirical Balanced Truncation . . . 12

3 ERK activation model **14**

4 Combined algorithm overview **14**
4.1 Importing SBML . . . 14
4.2 Nondimensionalisation . . . 15
4.3 Conservation analysis & application . . . 16
4.4 Proper lumping routine . . . 17
4.5 Empirical balanced truncation routine . . . 19

1 Overview of model reduction methods

1.1 The Galerkin projection

Methods of model reduction can be considered as a projection of the state-variables to a lower dimensional subspace $\mathcal{V}: \ \dim(\mathcal{V}) = \tilde{n}$ of the original phase-space, within which some relevant set of the system's trajectories can be adequately approximated. Mathematically, applying such a projection to obtain a reduced dynamical system is underpinned by the Galerkin projection [1] which will be introduced here.

Consider a basis B of the subspace $\mathcal{V}$ such that $B = [\boldsymbol{b}_1, \ldots, \boldsymbol{b}_{\tilde{n}}] \in \mathbb{R}^{n\times\tilde{n}}$. Assuming B has been selected such that it provides an adequately accurate approximation of the original states $\boldsymbol{x}(t)$ within the subspace $\mathcal{V}$, then

$$\boldsymbol{x}(t) \approx B\tilde{\boldsymbol{x}}(t) \tag{1}$$

with $\tilde{\boldsymbol{x}}(t) \in \mathbb{R}^{\tilde{n}}$ representing the reduced set of state-variables. Substituting this approximation into the stoichiometric model form yields

$$B\frac{\mathrm{d}\tilde{\boldsymbol{x}}}{\mathrm{d}t} = \boldsymbol{f}(B\tilde{\boldsymbol{x}}(t)) + \sum_{i=1}^{l} \boldsymbol{g}_i(B\tilde{\boldsymbol{x}}(t))u_i(t) + \boldsymbol{\rho}(t) \tag{2}$$

where it is assumed that B is time-invariant. Additionally, $\boldsymbol{\rho}(t) \in \mathbb{R}^n$ is termed the residual and addresses the discrepancy emerging from the fact that $B\tilde{\boldsymbol{x}}$ is typically not an exact solution of the system for all times.

Now let $\mathcal{W}$ represent a subspace that is orthogonal to the residual $\boldsymbol{\rho}(t)$ with a basis $C \in \mathbb{R}^{n\times\tilde{n}}$ such that $C^{\intercal}\boldsymbol{\rho}(t) = 0$. Hence, left multiplying equation (2) by $C^{\intercal}$ produces

$$C^{\intercal}B\frac{\mathrm{d}\tilde{\boldsymbol{x}}}{\mathrm{d}t} = C^{\intercal}\boldsymbol{f}(B\tilde{\boldsymbol{x}}(t)) + \sum_{i=1}^{l} C^{\intercal}\boldsymbol{g}_i(B\tilde{\boldsymbol{x}}(t))u_i(t) \tag{3}$$

Assuming $C^{\intercal}B$ is non-singular, this finally leads to a reduced dynamical system of the form

$$\frac{\mathrm{d}\tilde{\boldsymbol{x}}}{\mathrm{d}t} = \left(C^{\intercal}B\right)^{-1}C^{\intercal}\boldsymbol{f}(B\tilde{\boldsymbol{x}}(t)) + \sum_{i=1}^{l}\left(C^{\intercal}B\right)^{-1}C^{\intercal}\boldsymbol{g}_i(B\tilde{\boldsymbol{x}}(t))u_i(t). \tag{4}$$

This simplification of a dynamical system to a lower dimensional subspace is known as the Petrov-Galerkin projection. In the special case where $B = C$ it is known simply as the Galerkin projection. In that case

$$\left(B^{\intercal}B\right)^{-1}B^{\intercal} = \bar{B} \tag{5}$$

Such that $\bar{B}$ is a generalised left inverse of B and $\bar{B}B = I_{\tilde{n}}$ (the $\tilde{n}$ dimensional identity matrix).

Whilst the explanation given above provides an account of how to apply a Petrov-Galerkin projection, it does not provide a methodology for finding suitable bases B and C for a given model.

1.2 Conservation analysis

To understand the nature of conservation relations and how they might be found computationally, first note that a common means for representing the network structure underlying a system of chemical equations is that of the stoichiometry matrix. This is an $n \times m$ matrix S, with each of the rows corresponding to a single species and each of the columns to a reaction. The matrix is populated such that its entries s_{ij} give the net value of the stoichiometric coefficients (product minus reactant) of the i-th species in the j-th reaction. If the concentration of a particular species is not affected by a reaction the corresponding entry is populated with a 0. Hence, the sign of the entry indicates whether the species is a net reactant or a net product in the relevant reaction. A positive sign implies that the species is a product (i.e. the number of molecules is increased by the reaction), whilst a negative sign indicates that the species is a reactant (i.e. the number of molecules is decreased).

This matrix can be considered as mapping the vector of reaction rates $\boldsymbol{v}(\boldsymbol{x}(t))$ to the change in species concentration. Hence it is possible to represent the system of ODEs in the form

$$\dot{\boldsymbol{x}}(t) = S\boldsymbol{v}(\boldsymbol{x}(t)). \tag{6}$$

Now turning to conservation analysis and following the ideas outlined by Reder [2], the existence of conservation relations in a model implies that

$$\Gamma\dot{\boldsymbol{x}}(t) = 0 \tag{7}$$

where Γ is an $h \times n$ matrix that will be referred to here as the conservation matrix, the rows of which represent the linear combinations of species that are constant in time. Alternatively, by integration,

$$\Gamma\boldsymbol{x}(t) = \boldsymbol{c}, \tag{8}$$

the h individual elements of which are known as conservation relations, with $\boldsymbol{c} \in \mathbb{R}^h$ representing a set of constants known as conserved values.

It is hence possible to express a model containing conservation relations in the form of a system of differential algebraic equations (DAEs). To see this, first partition $\boldsymbol{x}$ into two subsets: $\boldsymbol{x}_d$ an h dimensional subset of the species with each element corresponding to a single species involved in a given conservation relation termed the dependent species. And $\boldsymbol{x}_i$ an $n-h$ dimensional subset accommodating all remaining state-variables, termed the independent species, such that

$$\boldsymbol{x}(t) = \begin{bmatrix} \boldsymbol{x}_d(t) \\ \boldsymbol{x}_i(t) \end{bmatrix}. \tag{9}$$

Then from equation (8)

$$\Gamma \begin{bmatrix} \boldsymbol{x}_d(t) \\ \boldsymbol{x}_i(t) \end{bmatrix} = \boldsymbol{c}. \tag{10}$$

This is a system of linear equations and hence if Γ is expressed in reduced row echelon form, such that

$$\Gamma = [I_h \; N_0] \tag{11}$$

with I_h representing the h dimensional identity matrix and N_0 a $h \times (n-h)$ matrix, it becomes apparent that

$$\boldsymbol{x}_d(t) = \boldsymbol{c} - N_0 \boldsymbol{x}_i(t). \tag{12}$$

This implies that the subset of dependent species $\boldsymbol{x}_d$ can be eliminated from the governing system of ODEs by substituting in the appropriate element of equation (12). Hence, given the stoichiometric form given in equation (6), a system exhibiting conservation relations can be expressed in the form of a semi-explicit system of DAEs, such that

$$\dot{\boldsymbol{x}}_i = S_i \boldsymbol{v}(\boldsymbol{x}_i(t)), \tag{13a}$$
$$\boldsymbol{x}_d(t) = N_0 \boldsymbol{x}_i(t) - \boldsymbol{c}, \tag{13b}$$

where equation (13b) has been exploited in equation (13a) to obtain a system of ODEs such that state-variables $\boldsymbol{x}_d$ are no longer explicitly given. Additionally, S_i here represents the rows of the stoichiometric matrix corresponding to the independent state-variables $\boldsymbol{x}_i$.

Obtaining the conservation matrix Γ, particularly for large systems, is often not feasible from simple inspection. To understand a more algorithmic approach for obtaining this matrix, begin by recalling the stoichiometric form of a model. Decomposing the stoichiometric matrix via the same partition as the set of species leads to the system

$$\begin{pmatrix} \dot{\boldsymbol{x}}_d(t) \\ \dot{\boldsymbol{x}}_i(t) \end{pmatrix} = \begin{pmatrix} S_d \\ S_i \end{pmatrix} \boldsymbol{v}(\boldsymbol{x}_d(t), \boldsymbol{x}_i(t)). \tag{14}$$

However, via differentiation of equation (12)

$$\dot{\boldsymbol{x}}_d(t) = -N_0 \dot{\boldsymbol{x}}_i(t) = -N_0 S_i \boldsymbol{v}(\boldsymbol{x}_d(t), \boldsymbol{x}_i(t)). \tag{15}$$

Hence, $S_d = -N_0 S_i$ and therefore each conservation relation can be seen as corresponding to a linear dependency in the stoichiometry matrix. As such, conservation relations can be found by seeking the left null space Z_n of S (i.e. via finding the null space of S^T) such that

$$Z_n = \left\{ \boldsymbol{z} \in \mathbb{R}^n \,|\, S^{\mathsf{T}} \boldsymbol{z} = \boldsymbol{0} \right\}, \tag{16}$$

and hence $Z_n^{\mathsf{T}} S = \boldsymbol{0}$. This implies that

$$Z_n^{\mathsf{T}} S \boldsymbol{v}\left(\dot{\boldsymbol{x}}(t)\right) = \boldsymbol{0} = Z_n^{\mathsf{T}} \dot{\boldsymbol{x}}(t) \tag{17}$$

and therefore via comparison to equation (7) it is clear that

$$Z_n^{\intercal} = \Gamma, \tag{18}$$

such that the conservation matrix is equal to the transpose of the left null space of the stoichiometry matrix. Hence, a more mathematically rigorous approach for finding conservation relations has been provided.

For very large systems (i.e. $n > 100$) $S^{\intercal}$ will be a large and, typically, sparse matrix. As a result, solving the system of linear equations

$$S^{\intercal}\boldsymbol{z} = \mathbf{0} \tag{19}$$

for each conservation relation may not always be numerically stable or efficient under traditional approaches such as Gaussian elimination. Therefore, the combined algorithm employs QR factorisation via Householder reflections, as is discussed in the main text.

1.3 Empirical balanced truncation

The description of empirical balanced truncation provided here is based upon the procedure given by Hahn and Edgar [3]. The aim is to construct two covariance matrices, known as the empirical controllability and observability Gramians, via repeated simulations of the system under perturbations of the input and the initial conditions respectively. As in the linear case, the empirical controllability Gramian provides information on how changes in the input will alter the state of the system. The empirical observability Gramian provides information on the total magnitude of the output that any given initial condition of the system can produce.

To construct the empirical Gramians, first recall that n refers to the original number of state-variables and l to the number of inputs. Additionally, we here define s to refer to the number of perturbation magnitudes and r to refer to the number of perturbation directions. Given this, we now define the following terms:

- $\mathbb{E}_C^n = \{e_{C,1}, \ldots, e_{C,l}\}$ representing the set of standard unit vectors in the input space $\mathbb{R}^l$;
- $\mathbb{E}_O^n = \{e_{O,1}, \ldots, e_{O,n}\}$ representing the set of standard unit vectors in the state space $\mathbb{R}^n$;
- $\mathbb{P}_C^r = \{P_{C,1}, \ldots, P_{C,r}\}$, a set of r orthogonal ($P_{C,i}^{\intercal} P_{C,i} = I,\ \forall i$) $l \times l$ matrices representing the perturbation directions explored for the set of inputs;
- $\mathbb{P}_O^r = \{P_{O,1}, \ldots, P_{O,r}\}$, a set of r orthogonal ($P_{O,i}^{\intercal} P_{O,i} = I,\ \forall i$) $n \times n$ matrices representing the perturbation directions explored for the set of initial conditions; and
- $\mathbb{M} = \{c_1, \ldots, c_s\}$ is a set of s positive, real constants representing the various magnitudes of the perturbations;

The Gramians are then computed using finite trajectory samples measured at regularly placed, discrete time points separated by a sampling interval Δt. Additionally q is defined to be the number of points in the sample. To ensure that accurate empirical Gramians are obtained it is required that the system is approximately at equilibrium at some time $t^* \leq q\Delta t$. Using these discrete time point samples the controllability Gramian is defined as

$$\mathcal{P} := \sum_{h=1}^{r} \sum_{m=1}^{s} \sum_{i=1}^{l} \frac{1}{rsc_m^2} \sum_{k=0}^{q} \psi_k^{ihm} \Delta t, \tag{20}$$

where $\psi^{ihm}(t) = \left(\mathbf{x}^{ihm}(t) - \mathbf{x}_{ss}^{ihm}\right)\left(\mathbf{x}^{ihm}(t) - \mathbf{x}_{ss}^{ihm}\right)^{\intercal}$ and $x^{ihm}(t)$ is the state or condition of system at time t corresponding to the perturbed input $\boldsymbol{u}(t) = c_m P_{C,h} e_{C,i} \delta(t) + \boldsymbol{u}_0$. Additionally, $\boldsymbol{u}_0$ is the steady-state

or unperturbed level of the input and $\mathbf{x}_{ss}^{ihm}$ represents the steady-state of the system produced under the perturbed input.

Meanwhile, the observability Gramian is similarly defined as

$$\mathcal{Q} := \sum_{h=1}^{r} \sum_{m=1}^{s} \frac{1}{rsc_m^2} \sum_{k=0}^{q} P_{O,h} \Phi_k^{hm} P_{O,h}^{\intercal} \Delta t, \tag{21}$$

where $\left\{\Phi^{hm}\right\}_{ij} = \left(\boldsymbol{y}^{ihm}(t) - \boldsymbol{y}_{ss}^{ihm}\right)^{\intercal} \left(\boldsymbol{y}^{jhm}(t) - \boldsymbol{y}_{ss}^{jhm}\right)$ and $\boldsymbol{y}^{ihm}(t)$ is the output of the system corresponding to the perturbed initial condition $\boldsymbol{x}^{ihm}(0) = c_m P_{O,h} e_{O,i} + \boldsymbol{x}_0$, with $\boldsymbol{x}_0$ representing the original, unperturbed initial condition of the system. Additionally, $\boldsymbol{y}_{ss}^{ihm}$ represents the steady-state of the output under this perturbed initial condition.

Once the Gramians have been computed the aim is to construct a balancing transformation of the state-variables which also acts to equalise and diagonalise the Gramians. This can be achieved by following the approach outlined in Methods Section of the main text.

2 Chemotaxis model summary

The specific model of chemotactic signalling analysed in the main text was originally described in Tindall et al. [4]. It is an 11 dimensional, nonlinear model describing a system of 12 biochemical reactions given by

$$\text{CheA} \overset{k_1(L)}{\rightarrow} \text{CheA}_P, \tag{22a}$$

$$\text{CheA}_P + \text{CheY} \underset{k_{-2}}{\overset{k_2}{\rightleftarrows}} \text{CheA}_P \cdot \text{CheY} \underset{k_{-7}}{\overset{k_7}{\rightleftarrows}} \text{CheA} \cdot \text{CheY}_P \underset{k_{-8}}{\overset{k_8}{\rightleftarrows}} \text{CheA} + \text{CheY}_P, \tag{22b}$$

$$\text{CheA} \cdot \text{CheY}_P \overset{k_6}{\rightarrow} \text{CheA} \cdot \text{CheY} \overset{k_{10}(L)}{\rightarrow} \text{CheA}_P \cdot \text{CheY}, \tag{22c}$$

$$\text{CheA} + \text{CheY} \underset{k_{-9}}{\overset{k_9}{\rightleftarrows}} \text{CheA} \cdot \text{CheY}, \tag{22d}$$

$$\text{CheY}_P + \text{CheZ} \underset{k_{-11}}{\overset{k_{11}}{\rightleftarrows}} \text{CheY}_P \cdot \text{CheZ} \overset{k_{12}}{\rightarrow} \text{CheY} + \text{CheZ}, \tag{22e}$$

$$\text{CheB}_P \overset{k_5}{\rightarrow} \text{CheB}, \tag{22f}$$

$$\text{CheY}_P \overset{k_6}{\rightarrow} \text{CheY}, \tag{22g}$$

$$\text{CheA}_P + \text{CheB} \overset{k_3}{\rightarrow} \text{CheA} + \text{CheB}_P. \tag{22h}$$

Whilst a more detailed account of this network can be found in the original paper, the model can be broadly understood as follows: Here, CheA represents a histidine kinase whose rate of autophosphorylation is modulated via extracellular attractant-receptor binding (in this case represented in equations (22a) and (22c) by the reaction rates $k_1(L)$ and $k_{10}(L)$, where L represents the concentration of extracellular ligand). An increase in attractant binding results in a decreased rate of phosphorylation of CheA. Two response regulator proteins (here represented by CheB and CheY and their interactions in equations (22b), (22d), and (22h)) compete to bind with protein CheA. Once bound CheA_P will transfer its phosphoryl group, hence providing the only means of phosphorylation for proteins CheB and CheY. In the case of protein CheY this phosphotransfer is also reversible via the reactions given in equation (22c). After they have been phosphorylated both response regulator proteins steadily auto-dephosphorylate as given by equations (22f) and (22g). In the case of protein CheY, however, this process can be greatly accelerated by forming a complex with the phosphatase CheZ as described by equation (22e). Note that it is the concentration of phosphorylated protein CheY that regulates the process of chemotaxis by binding with parts of the

flagellar motor complex (the process is not explicitly described in this model). The model differentiates itself somewhat from previously published approaches in that explicitly describes the intermediary complexes involved in the phosphorylation of proteins CheB and CheY.

Employing the Law of Mass Action, chemical equations (22) can be modelled by the following system of ODEs

$$\frac{\mathrm{d}x_1(t)}{\mathrm{d}t} = -k_1u(t)x_1(t) + k_3x_2(t)x_{10}(t) + k_8x_4(t) - k_{-8}x_1(t)x_8(t) - k_9x_1(t)x_7(t) + k_{-9}x_5(t), \tag{23a}$$

$$\frac{\mathrm{d}x_2(t)}{\mathrm{d}t} = k_1u(t)x_1(t) - k_2x_2(t)x_7(t) + k_{-2}x_3(t) - k_3x_2(t)x_{10}(t), \tag{23b}$$

$$\frac{\mathrm{d}x_3(t)}{\mathrm{d}t} = k_2x_2(t)x_7(t) - k_{-2}x_3(t) - k_7x_3(t) + k_{-7}x_4(t) + k_1u(t)x_5(t), \tag{23c}$$

$$\frac{\mathrm{d}x_4(t)}{\mathrm{d}t} = -k_6x_4(t) + k_7x_3(t) - k_{-7}x_4(t) - k_8x_4(t) + k_{-8}x_1(t)x_8(t), \tag{23d}$$

$$\frac{\mathrm{d}x_5(t)}{\mathrm{d}t} = k_6x_4(t) + k_9x_1(t)x_7(t) - k_{-9}x_5(t) - k_1u(t)x_5(t), \tag{23e}$$

$$\frac{\mathrm{d}x_6(t)}{\mathrm{d}t} = k_{11}x_8(t)x_9(t) - k_{-11}x_6(t) - k_{12}x_6(t), \tag{23f}$$

$$\begin{aligned}\frac{\mathrm{d}x_7(t)}{\mathrm{d}t} &= -k_2x_2(t)x_7(t) + k_{-2}x_3(t) - k_9x_1(t)x_7(t)\\ &\quad + k_{-9}x_5(t) + k_{12}x_6(t) + k_6x_8(t),\end{aligned} \tag{23g}$$

$$\frac{\mathrm{d}x_8(t)}{\mathrm{d}t} = k_8x_4(t) - k_{-8}x_1(t)x_8(t) - k_{11}x_8(t)x_9(t) + k{-}11x_6(t) - k_6x_8(t), \tag{23h}$$

$$\frac{\mathrm{d}x_9(t)}{\mathrm{d}t} = -k_{11}x_8(t)x_9(t) + k_{-11}x_6(t) + k_{12}x_6(t), \tag{23i}$$

$$\frac{\mathrm{d}x_{10}(t)}{\mathrm{d}t} = -k_3x_2(t)x_{10}(t) + k_5x_{11}(t), \tag{23j}$$

$$\frac{\mathrm{d}x_{11}(t)}{\mathrm{d}t} = k_3x_2(t)x_{10}(t) - k_5x_{11}(t), \tag{23k}$$

where $x_1(t) = [\text{CheA}]$, $x_2(t) = [\text{CheA}_P]$, $x_3(t) = [\text{CheA}_P \cdot \text{CheY}]$, $x_4(t) = [\text{CheA} \cdot \text{CheY}_P]$, $x_5(t) = [\text{CheA} \cdot \text{CheY}]$, $x_6(t) = [\text{CheY}_P \cdot \text{CheZ}]$, $x_7(t) = [\text{CheY}]$, $x_8(t) = [\text{CheY}_P]$, $x_9(t) = [\text{CheZ}]$, $x_{10}(t) = [\text{CheB}]$, and $x_{11}(t) = [\text{CheB}_P]$. The initial condition employed in analysing this system was

$$\begin{aligned} &x_1(0) = 2.579\mu M, && x_2(0) = 3.084\mu M, && x_3(0) = 0.079\mu M, && x_4(0) = 0.728\mu M,\\ &x_5(0) = 1.430\mu M, && x_6(0) = 2.962\mu M, && x_7(0) = 1.383\mu M, && x_8(0) = 3.118\mu M,\\ &x_9(0) = 0.838\mu M, && x_{10}(0) = 0.004\mu M, && x_{11}(0) = 0.276\mu M. \end{aligned}$$

The specific parameterisation employed for this model can be found in Table 1.

To understand the input u, note that receptor binding of the extracellular chemotactic ligand (denoted by L) is described by Michealis-Menten kinetics, such that

$$k_1(L) = k_{10}(L) = k_1\left(1 - \frac{L^h}{K^h + L^h}\right), \tag{24}$$

where K is a Michaelis-Menten constant and h is a Hill-coefficient. For the sake of simplicity the input $u(t)$ is thus defined here to be

$$u(t) = 1 - \frac{L^h}{K^h + L^h} \tag{25}$$

such that $k_1(L) = k_{10}(L) = k_1u(t)$.

Table 1. The set of parameter values associated with the chemotaxis signalling pathway model in *E. coli* as detailed in [4].

Parameter	Value	Units
k_1	3.75	s^{-1}
k_2	2.50×10^6	$(Ms)^{-1}$
k_{-2}	15	s^{-1}
k_3	1.5×10^7	$(Ms)^{-1}$
k_5	0.7	s^{-1}
k_6	8.5×10^{-2}	s^{-1}
k_7	650	s^{-1}
k_{-7}	50	s^{-1}
k_8	250	s^{-1}
k_{-8}	2.08×10^7	$(Ms)^{-1}$
k_9	7.50×10^6	$(Ms)^{-1}$
k_{-9}	15	s^{-1}
k_{10}	3.75	s^{-1}
k_{11}	5.60×10^6	$(Ms)^{-1}$
k_{-11}	0.04	s^{-1}
k_{12}	4.90	s^{-1}
A_T	7.9	μM
B_T	0.28	μM
Y_T	9.7	μM
Z_T	3.8	μM
K	2.6	μM
h	1.2	

2.1 Reduction

In the following sections we will describe how the various steps of the combined model reduction algorithm were applied to the model of bacterial chemotaxis and reproduce the results it obtained.

2.1.1 Nondimensionalisaton

The algorithm initially randomly samples a selection of 50 combinations of the original parameters in order to compute a nondimensionalisation with the best numerical properties. This led to equations (26) with the associated parameters from Table 2,

$$\frac{\mathrm{d}z_1(\tau)}{\mathrm{d}\tau} = -\alpha_1 z_1(\tau)u(\tau) + \alpha_4 z_2(\tau)z_{10}(\tau) + z_4(\tau) - \alpha_9 z_1(\tau)z_8(\tau) - \alpha_{10} z_1(\tau)z_7(\tau) + \alpha_{11} z_5(\tau), \tag{26a}$$

$$\frac{\mathrm{d}z_2(\tau)}{\mathrm{d}\tau} = \alpha_1 z_1(\tau)u(\tau) - \alpha_2 z_2(\tau)z_7(\tau) + \alpha_3 z_3(\tau) - \alpha_4 z_2(\tau)z_{10}(\tau), \tag{26b}$$

$$\frac{\mathrm{d}z_3(\tau)}{\mathrm{d}\tau} = \alpha_2 z_2(\tau)z_7(\tau) - \alpha_3 z_3(\tau) - \alpha_7 z_3(\tau) + \alpha_8 z_4(\tau) + \alpha_1 z_5(\tau)u(\tau), \tag{26c}$$

$$\frac{\mathrm{d}z_4(\tau)}{\mathrm{d}\tau} = -\alpha_6 z_4(\tau) + \alpha_7 z_3(\tau) - \alpha_8 z_4(\tau) - z_4(\tau) + \alpha_9 z_1(\tau)z_8(\tau), \tag{26d}$$

$$\frac{\mathrm{d}z_5(\tau)}{\mathrm{d}\tau} = \alpha_6 z_4(\tau) + \alpha_{10} z_1(\tau)z_7(\tau) - \alpha_{11} z_5(\tau) - \alpha_1 z_5(\tau)u(\tau), \tag{26e}$$

$$\frac{\mathrm{d}z_6(\tau)}{\mathrm{d}\tau} = \alpha_{13} z_8(\tau)z_9(\tau) - \alpha_{14} z_6(\tau) - \alpha_{16} z_6(\tau), \tag{26f}$$

$$\frac{\mathrm{d}z_7(\tau)}{\mathrm{d}\tau} = -\alpha_2 z_2(\tau)z_7(\tau) + \alpha_3 z_3(\tau) - \alpha_{10} z_1(\tau)z_7(\tau) + \alpha_{11} z_5(\tau) + \alpha_{17} z_6(\tau) + \alpha_6 z_8(\tau), \tag{26g}$$

$$\frac{\mathrm{d}z_8(\tau)}{\mathrm{d}\tau} = z_4(\tau) - \alpha_9 z_1(\tau)z_8(\tau) - \alpha_{12} z_8(\tau)z_9(\tau) + \alpha_{15} z_6(\tau) - \alpha_6 z_8(\tau), \tag{26h}$$

$$\frac{\mathrm{d}z_9(\tau)}{\mathrm{d}\tau} = -\alpha_{13} z_8(\tau)z_9(\tau) + \alpha_{14} z_6(\tau) + \alpha_{16} z_6(\tau), \tag{26i}$$

$$\frac{\mathrm{d}z_{10}(\tau)}{\mathrm{d}\tau} = -\alpha_4 z_2(\tau)z_{10}(\tau) + \alpha_5 z_{11}(\tau), \tag{26j}$$

$$\frac{\mathrm{d}z_{11}(\tau)}{\mathrm{d}\tau} = \alpha_4 z_2(\tau)z_{10}(\tau) - \alpha_5 z_{11}(\tau). \tag{26k}$$

Where $t = k_8\tau$, $x_1(t) = B_T z_1(\tau)$, $x_2(t) = B_T z_2(\tau)$, $x_3(t) = B_T z_3(\tau)$, $x_4(t) = B_T z_4(\tau)$, $x_5(t) = B_T z_5(\tau)$, $x_6(t) = A_T z_6(\tau)$, $x_7(t) = B_T z_7(\tau)$, $x_8(t) = B_T z_8(\tau)$, $x_9(t) = A_T z_9(\tau)$, $x_{10}(t) = B_T z_{10}(\tau)$, and $x_{11}(t) = B_T z_{11}(\tau)$.

2.1.2 Conservation Analysis

Algorithm selects species CheA, CheA · CheY, CheZ, and CheB_P for elimination via the application of conservation relations.This can be achieved by substituting the following algebraic equations into the set of nondimensionalised differential equations represented by equations (26),

$$[\text{CheB}_P] = B_T - [\text{CheB}] \tag{27}$$

$$[\text{CheZ}_P] = Z_T - [\text{CheY}_P \cdot \text{CheZ}] \tag{28}$$

$$[\text{CheA} \cdot \text{CheY}] = Y_T - [\text{CheY}] - [\text{CheY}_P] - [\text{CheA}_P \cdot \text{CheY}] - [\text{CheA} \cdot \text{CheY}_P] - [\text{CheY}_P \cdot \text{CheZ}] \tag{29}$$

$$[\text{CheA}] = A_T - Y_T + [\text{CheY}_P] + [\text{CheY}] + [\text{CheY}_P \cdot \text{CheZ}] - [\text{CheA}_P] \tag{30}$$

Table 2. The set of nondimensionalised parameter values associated with the chemotaxis signalling pathway model in *E. coli* as defined by equation (26).

Parameter	Original Expression	Value
α_1	$\frac{k_1}{k_8}$	0.0150
α_2	$\frac{k_2 B_T}{k_8}$	0.0028
α_3	$\frac{k_{-2}}{k_8}$	0.06
α_4	$\frac{k_3 B_T}{k_8}$	0.0168
α_5	$\frac{k_5}{k_8}$	0.0028
α_6	$\frac{k_6}{k_8}$	3.4×10^{-4}
α_7	$\frac{k_7}{k_8}$	2.6
α_8	$\frac{k_{-7}}{k_8}$	0.2
α_9	$\frac{k_{-8} B_T}{k_8}$	0.0233
α_{10}	$\frac{k_9 B_T}{k_8}$	0.0084
α_{11}	$\frac{k_{-9}}{k_8}$	0.06
α_{12}	$\frac{k_{11} A_T}{k_8}$	0.1770
α_{13}	$\frac{k_{11} B_T}{k_8}$	0.0063
α_{14}	$\frac{k_{-11}}{k_8}$	1.6×10^{-4}
α_{15}	$\frac{k_{-11} A_T}{k_8 B_T}$	0.0045
α_{16}	$\frac{k_{12}}{k_8}$	0.0196
α_{17}	$\frac{k_{12} A_T}{k_8 B_T}$	0.5530

Table 3. An additional set of nondimensionalised parameter values associated with the chemotaxis signalling pathway model in *E. coli* as defined by equation (31).

Parameter	Original Expression	Value
β_1	$\frac{A_T}{B_T}$	28.21
β_2	$\frac{Y_T}{B_T}$	34.64
β_3	$\frac{Z_T}{B_T}$	13.57
β_4	$\frac{Z_T}{A_T}$	0.48

This yields the following system

$$\frac{\mathrm{d}z_2(\tau)}{\mathrm{d}\tau} =(z_7(\tau) + z_8(\tau) - z_2(\tau) + \beta_1 z_6(\tau) + \beta_1 - \beta_2)\alpha_1 u(\tau) - \alpha_2 z_2(\tau) z_7(\tau) + \alpha_3 z_3(\tau) - \alpha_4 z_2(\tau) z_{10}(\tau), \tag{31a}$$

$$\frac{\mathrm{d}z_3(\tau)}{\mathrm{d}\tau} =(\beta_2 - z_7(\tau) - z_3(\tau) - z_4(\tau) - \beta_1 z_6(\tau) - z_8(\tau))\alpha_1 u(\tau) + \alpha_2 z_2(\tau) z_7(\tau) - \alpha_3 z_3(\tau) - \alpha_7 z_3(\tau) + \alpha_8 z_4(\tau), \tag{31b}$$

$$\frac{\mathrm{d}z_4(\tau)}{\mathrm{d}\tau} = - \alpha_6 z_4(\tau) + \alpha_7 z_3(\tau) - \alpha_8 z_4(\tau) - z_4(\tau) + (z_7(\tau) + z_8(\tau) - z_2(\tau) + \beta_1 z_6(\tau) + \beta_1 - \beta_2)\alpha_9 z_8(\tau), \tag{31c}$$

$$\frac{\mathrm{d}z_6(\tau)}{\mathrm{d}\tau} =(\beta_3 - z_6(\tau))\alpha_{13} z_8(\tau) - \alpha_{14} z_6(\tau) - \alpha_{16} z_6(\tau), \tag{31d}$$

$$\frac{\mathrm{d}z_7(\tau)}{\mathrm{d}\tau} = - \alpha_2 z_2(\tau) z_7(\tau) + \alpha_3 z_3(\tau) + \alpha_{17} z_6(\tau) + \alpha_6 z_8(\tau) - (z_7(\tau) + z_8(\tau) - z_2(\tau) + \beta_1 z_6(\tau) + \beta_1 - \beta_2)\alpha_{10} z_7(\tau) + \alpha_{11}(\beta_2 - z_7(\tau) - z_3(\tau) - z_4(\tau) - \beta_1 z_6(\tau) - z_8(\tau)), \tag{31e}$$

$$\frac{\mathrm{d}z_8(\tau)}{\mathrm{d}\tau} =z_4(\tau) + \alpha_{15} z_6(\tau) - \alpha_6 z_8(\tau) + (z_7(\tau) + z_8(\tau) - z_2(\tau) + \beta_1 z_6(\tau) + \beta_1 - \beta_2)\alpha_9 z_8(\tau) - (z_6(\tau) - \beta_4)\alpha_{12} z_8(\tau), \tag{31f}$$

$$\frac{\mathrm{d}z_{10}(\tau)}{\mathrm{d}\tau} = - \alpha_4 z_2(\tau) z_{10}(\tau) - \alpha_5 z_{10}(\tau) + \alpha_5, \tag{31g}$$

$$\tag{31h}$$

with the additional nondimensional parameters given in Table 3

2.1.3 Lumping

The algorithm then proceeds to reduce the system via proper lumping to a 5 dimensional form. In this section we will symbolically reproduce this reduced model. Through the forward selection procedure described in the main text, the algorithm determines that the optimal scheme is to lump together state-variables $z_3(\tau)$, $z_4(\tau)$, and $z_8(\tau)$ as one. These variables correspond to the original species $\mathrm{CheA}_P \cdot \mathrm{CheY}$, $\mathrm{CheA} \cdot \mathrm{CheY}_P$, and CheY_P, respectively.

If we assume a vector of state-variables, such that $\boldsymbol{y}(\tau) = [y_2(\tau), y_3(\tau), y_4(\tau), y_6(\tau), y_7(\tau), y_8(\tau), y_{10}(\tau)]^\intercal$,

then this scheme corresponds to the lumping matrix

$$L = \begin{bmatrix} 1 & 0 & 0 & 0 & 0 & 0 & 0 \\ 0 & 1 & 0 & 0 & 0 & 0 & 0 \\ 0 & 0 & 1 & 1 & 0 & 1 & 0 \\ 0 & 0 & 0 & 0 & 1 & 0 & 0 \\ 0 & 0 & 0 & 0 & 0 & 0 & 1 \end{bmatrix}.$$

Hence we have a set of reduced state-variables $\tilde{\boldsymbol{y}}(\tau) = L\boldsymbol{y}(\tau)$ such that

$$\begin{bmatrix} \tilde{z}_1(\tau) \\ \tilde{z}_2(\tau) \\ \tilde{z}_3(\tau) \\ \tilde{z}_4(\tau) \\ \tilde{z}_5(\tau) \end{bmatrix} = \begin{bmatrix} z_2(\tau) \\ z_3(\tau) \\ z_4(\tau) + z_6(\tau) + z_8(\tau) \\ z_7(\tau) \\ z_{10}(\tau) \end{bmatrix}$$

Following the general descriptions provided in the main text it is now necessary to construct a generalised right inverse $\bar{L}$ of the lumping matrix prior to application of the Galerkin projection. Such a matrix can be constructed by computing

$$\bar{L} = XL^{\intercal} \left(LXL^{\intercal}\right)^{-1}, \tag{32}$$

where X represents a diagonal matrix, typically containing steady-state values of the system. In the case of the nondimensionalised model described above, this would yield a matrix of the form

$$\bar{L} = \begin{bmatrix} 1 & 0 & 0 & 0 & 0 \\ 0 & 1 & 0 & 0 & 0 \\ 0 & 0 & \frac{z_4^*}{z_4^*+z_6^*+z_8^*} & 0 & 0 \\ 0 & 0 & \frac{z_6^*}{z_4^*+z_6^*+z_8^*} & 0 & 0 \\ 0 & 0 & 0 & 1 & 0 \\ 0 & 0 & \frac{z_8^*}{z_4^*+z_6^*+z_8^*} & 0 & 0 \\ 0 & 0 & 0 & 0 & 1 \end{bmatrix}.$$

Given these matrices it is then possible to construct a reduced description of the system's dynamical

Table 4. An additional set of parameter values associated with the lumped chemotaxis signalling pathway model in *E. coli* as defined by equation (33).

Parameter	Expression	Values Used
γ_1	$\frac{z_4^*}{z_4^*+z_6^*+z_8^*}$	0.0201
γ_2	$\frac{z_6^*}{z_4^*+z_6^*+z_8^*}$	0.1855
γ_3	$\frac{z_8^*}{z_4^*+z_6^*+z_8^*}$	0.7944

behaviour via the Galerkin projection, this yields the analytically reduced system

$$\begin{aligned}
\frac{\mathrm{d}\tilde{z}_1(\tau)}{\mathrm{d}\tau} &= (\tilde{z}_4(t) + \gamma_3\tilde{z}_3(t) - z_1(t) + \beta_1\gamma_2\tilde{z}_3(t) + \beta_1 - \beta_2)\alpha_1 u(t) \\
&\quad - \alpha_2\tilde{z}_1(t)\tilde{z}_4(t) + \alpha_3\tilde{z}_2(t) - \alpha_4\tilde{z}_1(t)\tilde{z}_5(t) && \text{(33a)} \\
\frac{\mathrm{d}\tilde{z}_2(\tau)}{\mathrm{d}\tau} &= (\beta_2 - \tilde{z}_4(t) - \tilde{z}_2(t) - \gamma_1\tilde{z}_3(t) - \beta_1\gamma_2\tilde{z}_3(t) - \gamma_3\tilde{z}_3(t))\alpha_1 u(t) + \alpha_2\tilde{z}_1(t)\tilde{z}_4(t) \\
&\quad - \alpha_3\tilde{z}_2(t) - \alpha_7\tilde{z}_2(t) + \alpha_8\gamma_1\tilde{z}_3(t) && \text{(33b)} \\
\frac{\mathrm{d}\tilde{z}_3(\tau)}{\mathrm{d}\tau} &= -\alpha_6\gamma_1\tilde{z}_3(t) + \alpha_7\tilde{z}_2(t) - \alpha_8\gamma_1\tilde{z}_3(t) + (\beta_3 - \gamma_2\tilde{z}_3(t))\alpha_{13}\gamma_3\tilde{z}_3(t) \\
&\quad - \alpha_{14}\gamma_2\tilde{z}_3(t) - \alpha_{16}\gamma_2\tilde{z}_3(t) + \alpha_{15}\gamma_2\tilde{z}_3(t) - \alpha_6\gamma_3\tilde{z}_3(t) - (\gamma_2\tilde{z}_3(t) - \beta_4)\alpha_{12}\gamma_3\tilde{z}_3(t) && \text{(33c)} \\
\frac{\mathrm{d}\tilde{z}_4(\tau)}{\mathrm{d}\tau} &= -\alpha_2\tilde{z}_1(t)\tilde{z}_4(t) + \alpha_3\tilde{z}_2(t) + \alpha_{17}\gamma_2\tilde{z}_3(t) \\
&\quad + \alpha_6\gamma_3\tilde{z}_3(t) - (\tilde{z}_4(t) + \gamma_3\tilde{z}_3(t) - \tilde{z}_1(t) + \beta_1\gamma_2\tilde{z}_3(t) + \beta_1 - \beta_2)\alpha_{10}\tilde{z}_4(t) \\
&\quad + \alpha_{11}(\beta_2 - \tilde{z}_4(t) - \tilde{z}_2(t) - \gamma_1\tilde{z}_3(t) - \beta_1\gamma_2\tilde{z}_3(t) - \gamma_3\tilde{z}_3(t)) && \text{(33d)} \\
\frac{\mathrm{d}\tilde{z}_5(\tau)}{\mathrm{d}\tau} &= -\alpha_4\tilde{z}_1(t)\tilde{z}_5(t) + \alpha_5\tilde{z}_{11}(t), && \text{(33e)} \\
& && \text{(33f)}
\end{aligned}$$

where the new parameters (γ_1, γ_2, and γ_3) are defined in Table 4. The specific values reported were calculated via the novel, averaged inverse approach that is outlined in the main manuscript.

2.1.4 Empirical Balanced Truncation

Given the system reduced via the application of nondimensionalisation, conservation and lumping described by equations (33) and the parameter sets defined by Tables 2, 3 and 4, it is now possible to apply empirical balanced truncation in order to achieve further reduction of this system following the combined model reduction algorithm.

To construct the empirical Gramians we employed simulated data for 100 distinct perturbations to both the unpertubed model input $u_0 = 1$ and each of the unperturbed system's initial conditions $\tilde{y}_0$, in all instances perturbations were uniformly sampled from of 0.2 to 1.8 times the parameter in question's original

or unperturbed values. Following the approach described in Section 1.3, this yielded the Gramians

$$\mathcal{P} = \begin{bmatrix} 2829.32 & -3560.27 & -4720.77 & -86.65 & 53.06 \\ -3560.27 & 5687.27 & 6741.01 & 99.49 & -48.78 \\ -4720.77 & 6741.01 & 8491.32 & 136.97 & -71.36 \\ -86.65 & 99.49 & 136.97 & 2.75 & -1.85 \\ 53.06 & -48.78 & -71.36 & -1.85 & 1.83 \end{bmatrix}$$

and

$$\mathcal{Q} = \begin{bmatrix} 4.01 & 1.81 & 1.44 & 53.41 & 1328.71 \\ 1.81 & 0.84 & 0.68 & 24.87 & 598.31 \\ 1.44 & 0.68 & 0.58 & 20.31 & 474.48 \\ 53.41 & 24.87 & 20.31 & 735.38 & 17680.31 \\ 1328.71 & 598.31 & 474.48 & 17680.31 & 440366.67 \end{bmatrix}.$$

Calculating the balancing transformation following the procedure given in the main text then yields

$$\mathcal{T} = \begin{bmatrix} 0.0678 & 0.0304 & 0.0240 & 0.8977 & 22.4730 \\ 0.0110 & 0.0315 & 0.0468 & 0.9450 & 2.9809 \\ -0.0128 & 0.0654 & -0.0667 & 0.3988 & -0.0943 \\ -0.0118 & -0.0249 & 0.0387 & -2.0207 & -0.8598 \\ 0.0923 & -0.0038 & 0.0264 & 1.7307 & 0.0016 \end{bmatrix}.$$

If we now seek to create the 2 dimensional reduced system described in the main text we first need to define reduced variables $\tilde{\boldsymbol{x}}(\tau) = [\tilde{x}_1(\tau), \tilde{x}_2(\tau)]^{\intercal}$. However, to increase the accuracy of this reduced model it should be residualised, such that for reconstructing the values of the set of nondimensionalised variables $\boldsymbol{y}(\tau)$ from this reduced model we have

$$\begin{bmatrix} \tilde{z}_2(\tau) \\ \tilde{z}_3(\tau) \\ \tilde{z}_4(\tau) \\ \tilde{z}_6(\tau) \\ \tilde{z}_7(\tau) \\ \tilde{z}_8(\tau) \\ \tilde{z}_{10}(\tau) \end{bmatrix} \approx \bar{L}T^{-1} \begin{bmatrix} \tilde{x}_1(\tau) \\ \tilde{x}_2(\tau) \\ -0.1298 \\ -0.5604 \\ 1.4330 \end{bmatrix}.$$

Given these state-variables, the reduced model is then

$$\frac{\mathrm{d}\tilde{x}_1(\tau)}{\mathrm{d}\tau} = 0.0185\tilde{x}_1(\tau)^2 - 0.202\tilde{x}_1(\tau)\tilde{x}_2(\tau) + 0.0736\tilde{x}_2(\tau)^2 + 4.74\times10^{-4}u(\tau)\tilde{x}_1(\tau) - 0.0052u(\tau)\tilde{x}_2(\tau) + 0.0117 + 0.0130u(\tau) + 0.0484\tilde{x}_1(\tau) + 0.128\tilde{x}_2(\tau) \quad (34a)$$

$$\frac{\mathrm{d}\tilde{x}_2(\tau)}{\mathrm{d}\tau} = 0.0026\tilde{x}_1(\tau)^2 - 0.0279\tilde{x}_1(\tau)\tilde{x}_2(\tau) + 0.0101\tilde{x}_2(\tau)^2 + 0.0010u(\tau)\tilde{x}_1(\tau) - 0.0092u(\tau)\tilde{x}_2(\tau) + 0.0026 + 0.0197u(\tau) + 0.0075\tilde{x}_1(\tau) + 0.0133\tilde{x}_2(\tau) \quad (34b)$$

with associated initial conditions $\tilde{x}_1(0) = 1.6775$ and $\tilde{x}_2(0) = 1.4560$.

3 ERK activation model

Due to the size of the ERK activation model it is not possible to provide the same level of detail into its reduction as was provided for the model of bacterial chemotaxis signalling. We have, however, provided a number of Matlab files that describe the form of the model at various stages of reduction along with its initial conditions, associated lumping matrices and their generalised inverses, the empirical gramians and the balancing transformation. Use of these files does require Matlab's Symbolic Math toolbox. All of this is made available in Additional file 2 - ERK Activation Reduction Files.

4 Combined algorithm overview

In this section a more detailed account of the combined model reduction algorithm is provided. Figure 1 depicts the high level steps of the overall algorithm. The following sections correspond to each of these steps and describe the specific process and implementation in Matlab by which they are achieved. This section provides a more detailed unpacking of the algorithm steps given in Figure 2 of the main text.

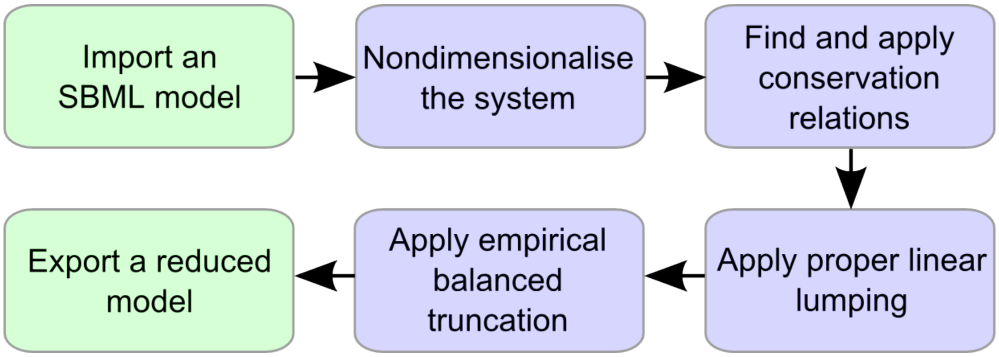

Figure 1. A shortened overall schematic of the combined model reduction algorithm. This is a submodule, schematic representation of the combined model reduction algorithm that will be detailed in sections 3.1 to 3.5.

4.1 Importing SBML

- Have the user define an SBML file representing the model, referred to here as 'SBML.xml'. Additionally, define the desired input values $\boldsymbol{u}$ and outputs $\boldsymbol{y}(t)$ in terms of the species given in the model. Finally, define a 'natural' or unperturbed input level $\boldsymbol{u}_0$ which corresponds to the initial condition of

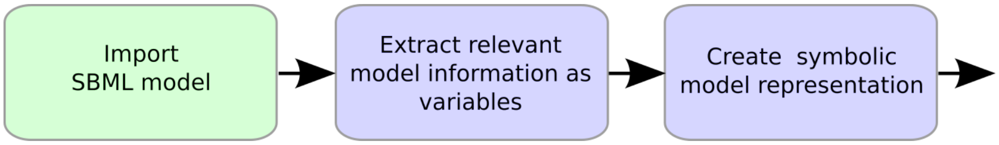

Figure 2. Import and symbolic conversion process in the combined model reduction algorithm.

the pathway before any intervention occurs (for example the concentration of an extracellular ligand considered as an input might be negligible until the administration of the particular drug of interest, hence $\boldsymbol{u}_0 = 0$).

- The algorithm uses the LibSBML Matlab library along with the open-source SBToolbox2 package to import SBML.xml as an SBmodel object named 'mod'.
- Commands from the SBToolbox2 package are then additionally used to extract specific model information from 'mod' as variables into the Matlab workspace. Specifically:
  - the set of species named 'Species';
  - the set of parameters named 'Params';
  - the set of reactions named 'Reacts'; and
  - the stoichiometry matrix named 'N'.
- The model is simulated with the initial conditions given in the SBML file and under the undisturbed input $\boldsymbol{u}_0$. Once steady-state has been reached this undisturbed equillibrium is defined to be the new initial condition of the system.
- A symbolic representation of the model named 'DiffFuncs' and its variables is then constructed via the Symbolic Matlab package. To achieve this the algorithm
  - defines a set of symbolic variables $x_i(t)$ of equal length to the vector Species;
  - creates a symbolic vector of reactions 'SReacts' by substituting Species$(i) = x_i(t)$ into Reacts;
  - Redefines the outputs $\boldsymbol{y}(t)$ in terms of these new state-variables $x_i(t)$ via substitution; and
  - computes DiffFuncs as the product of the stoichiometry matrix and SReacts, such that

$$\text{DiffFuncs} = N \cdot \text{SReacts}.$$

- DiffFuncs can then be converted to a form for simulation via the functions 'odeToVectorField' and 'matlabFunction'.
- For future reference, now simulate the system under all desired input values $\boldsymbol{u}$ and calculate the outputs $\boldsymbol{y}(t)$ under each. Store these results as the original output of the system 'OutO'.

4.2 Nondimensionalisation

- Begin by constructing a complete list of the parameters and their associated units:
  - construct a list of each monomial term contained within SReact named SR. Here the inbuilt 'strsplit' function is employed to create the list of monomials and $x_i(t) = 1$ is used for all i so as to access only the coefficient of each monomial;

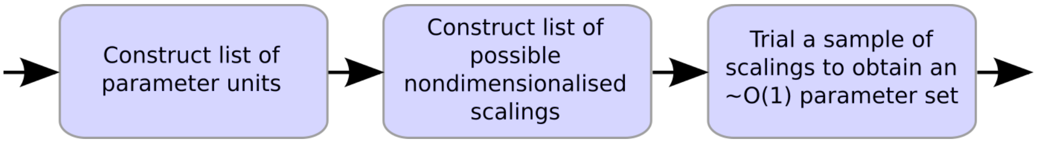

Figure 3. Nondimesionalisation process in the combined model reduction algorithm.

- additionally, compute the degree of each monomial within SR;
- use this list of degrees to inform the fundamental units associated with the kinetic rate coefficient of each monomial. Here use T to represent temporal units and M to represent mass based units; and
- if the conserved totals are not given in the predefined model parameters Params, then it is necessary to calculate them here via the steps outlined in Section 1.1.

- Substitute scaled variables, such that $x_i(t) = \alpha_i z_i(t)$ and $t = \beta\tau$, into the symbolic system of ODEs (DiffFuncs).
- Compute a list of the new coefficients of each monomial in this scaled system of ODEs (NDP).
- Use the list of units to create a list of possible nondimensionalisations for the system (the algorithm is restrained to $[\beta] = T$ such that β is the reciprocal of a $1/T$ kinetic rate coefficient and $[\alpha_i] = M$, $\forall i$ such that each α_i is chosen from the list of conserved total masses. Other nondimensionalisations for rate terms associated with nonlinear monomials will exist, but the algorithm is currently limited in this regard for the sake of computational efficiency).
- Even for modestly sized systems ($\sim$10's of ODEs) this will produce a very large list of possible nondimensionalisations. Hence, instead of evaluating them all, randomly select a sample of 50 possible nondimensionalisations (or use the total number if this is less than 50).
- Loop through this possible list of rescalings, substituting each into NDP with individual rescaling referred to as NDP(i).
  - For each possible rescaling calculate the range
    $\text{RNDP}(i) = \log_{10}(\max\{\text{NDP}(i)\}) - \log_{10}(\min\{\text{NDP}(i)\})$
  - Retain the rescaling that obtains the minimum value of RNDP(i).
- Substitute this nondimensionalisation into the symbolic system of ODEs to be regarded as the new model.

4.3 Conservation analysis & application

- Use an empirical Jacobian estimation algorithm around the initial conditions of the model. Here this is achieved using the SBtoolbox2 command 'SBjacobian'.
- Use this Jacobian estimate to approximate the speed of each reactant by simply using the diagonal elements. Rank the species in terms of these elements with larger values corresponding to faster species.
- Apply Matlab's inbuilt QR factorisation and reduced row echelon form algorithms to obtain the conservation relations for the model. Essentially this follows the approaches outlined in Section 1.2; apply QR factorisation 'qr' with pivoting to the stoichiometry matrix N to yield an orthogonal matrix

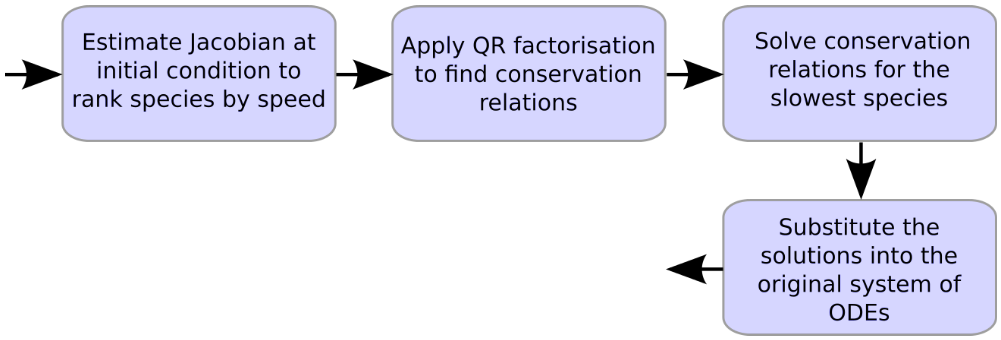

Figure 4. Conservation analysis and application process in the combined model reduction algorithm.

Q, an upper triangular matrix R, and a pivot matrix P. Use the 'rref' function to place R in reduced row echelon form, and finally re-transform this matrix under P to yield the previously defined conservation matrix Γ.

- Substitute in initial conditions to obtain values for conserved totals.
- Solve conservation relations for particular species, favouring those species approximated to be the slowest.
- Substitute solutions into DiffFuncs to give a reduced set of ODEs (dismissing those differential equations corresponding to state-variables that have been reduced to algebraic relations). This reduced set of ODEs is now treated as the new model.

4.4 Proper lumping routine

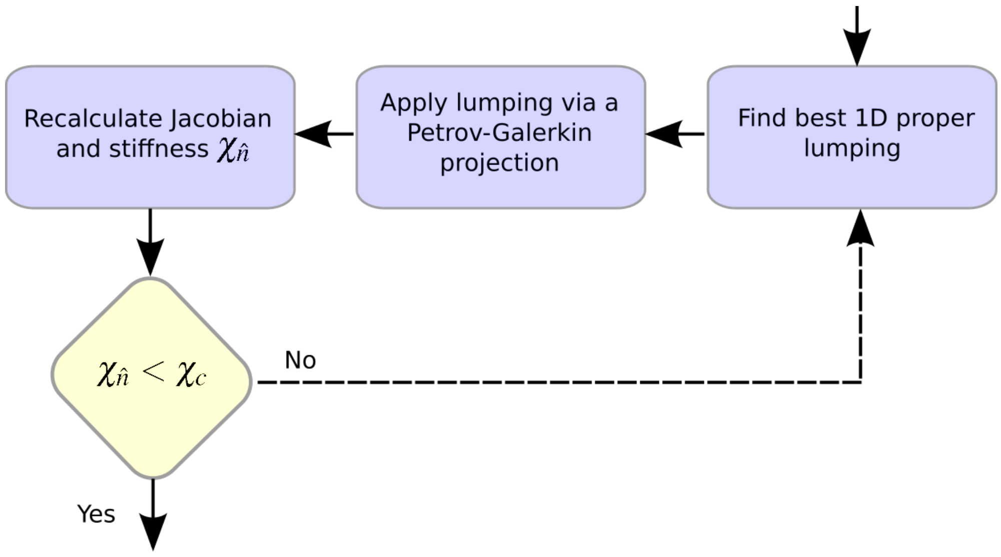

Figure 5. The proper lumping process in the combined model reduction algorithm.

- Begin by setting the 'previous lumping matrix' (L_{Prev}) and its generalised inverse ($\bar{L}_{\text{Prev}}$) equal to the n dimensional identity matrix I_n.
- Create table to store lumping results, named here as 'ResTab'.
- Enter the first loop which is designed at each step to reduce the model by one further dimension. Here, iterate for i from 1 to $(n-1)$.
  - Calculate the new reduced dimension $\tilde{n} = n - i$ and set the 'previous dimension' variable $n_{\text{prev}} = \tilde{n} + 1$.
  - Create a list of reduced symbolic variables $z_i(t)$, for $i = 1 \ldots \tilde{n}$.
  - Load previous lumping matrix (L_{Prev}) and its generalised inverse ($\bar{L}_{\text{Prev}}$).
  - Create a list of all possible proper, linear lumpings to dimension $\tilde{n}$ based upon a one dimensional lumping of L_{Prev} (there will be $\frac{1}{2}\left(n_{\text{prev}}\left[n_{\text{prev}} - 1\right]\right)$ possible lumpings to trial as it represents all possible pairings of the current n_{prev} state-variables). This list is referred to here as 'TempTab'.
  - Enter a parallelised second loop using Matlab's 'parfor' function. Here, each thread trials a possible lumping from TempTab and calculates the error and stiffness coefficient associated with it. Iterate for j from 1 to size(TempTab).
    * The one dimensional n_{Prev} to $\tilde{n}$ lumping matrix is defined by the j-th entry of TempTab, and is constructed here as L_{Temp}.
    * Then the new overall lumping matrix is computed as $L = L_{\text{Prev}} \cdot L_{\text{Temp}}$.
    * To trial both the steady-state and average lumping inverses, $\bar{L}$ and $\hat{L}$ respectively, now enter a final loop. Here, iterate for k from 1 to 2.
      · If $k = 1$: Define $X = \text{diag}\left(L_{\text{Prev}} \boldsymbol{x}^*_{\boldsymbol{u}_0}\right)$, such that X represents an $n_{\text{prev}} \times n_{\text{prev}}$ diagonal matrix of the steady-state values $\boldsymbol{x}^*_{\boldsymbol{u}_0}$ of the system under the unperturbed input.
      · Calculate $\bar{L}_{\text{Temp}} = X L_{\text{Temp}}^{\intercal} \left(L_{\text{Temp}} X L_{\text{Temp}}^{\intercal}\right)^{-1}$.
      · If $k = 2$: Use the novel average inverse defined in main text, such that $\bar{L}_{\text{Temp}} = \hat{L}$.
      · Now compute the overall lumping inverse such that $\bar{L} = \bar{L}_{\text{Temp}} \cdot \bar{L}_{\text{Prev}}$.
      · Construct the reduced system as $\dot{\boldsymbol{z}}(t) = L\boldsymbol{f}(\bar{L}\boldsymbol{z}(t))$ by substituting 'reconstructed' state-variables $\bar{L}\boldsymbol{z}$ into DiffFuncs and then lumping this vector of symbolic equations via L.
      · Simulate this reduced model and calculate error as compared with the output of the original system (OutO). Additionally, re-estimate Jacobian and hence the stiffness coefficient $\chi_{\tilde{n}}$. Record both the error and stiffness for the specific lumping in TempTab.
    * End the lumping inverses loop.
  - End parallelised loop.
  - Select the lumping regime within a small error bound of the minimum error with the lowest stiffness coefficient.
  - Set $L_{\text{Prev}} = L$ and $\bar{L}_{\text{Prev}} = \bar{L}$.
  - Record these 'optimal' error and stiffness results for reduction $\tilde{n}$ in ResTab.
- End main loop.
- Select the lowest error lumping below the threshold stiffness coefficient (set currently to $\chi_{\tilde{n}} < 250$ based upon numerical experimentation) and save the corresponding reduced model as the new system.
- Let n_L represent the new dimensionality of the model after lumping.

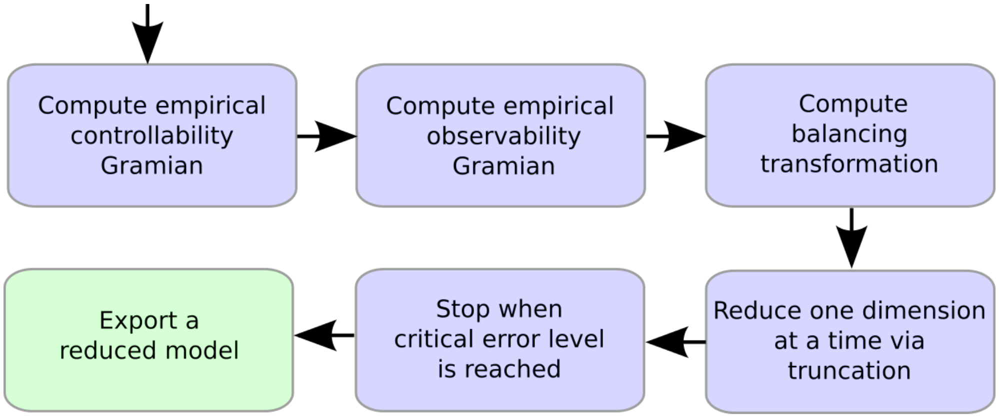

Figure 6. Empirical balanced truncation process in the combined model reduction algorithm.

4.5 Empirical balanced truncation routine

Compute the empirical controllability Gramian $\mathcal{P}$ following the approach outlined in Section 1.3:

- Define $\mathcal{P}$ as an empty $n_L \times n_L$ matrix.
- Define a list of s random input perturbation sizes (M). Here, typically 100 random sizes from 0 to the magnitude of $\boldsymbol{u}$ are selected.
- Define a list of r perturbation directions in input space. Here the algorithm currently employs $r = 2$ corresponding to an increase in all inputs and a decrease in all inputs. Additionally, define $P(h)$ to represent an $l \times l$ diagonal matrix of the h-th perturbation direction.
- Define a set of l unit vectors in input space (E). Recall that l simply represents the number of inputs in the model.
- Enter the first loop going through each of the perturbation sizes. This loop is parallelized using Matlab's 'parfor' function. Here, iterate through m from 1 to s.
  - Enter the second loop going through each of the perturbation directions. Here, iterate through h from 1 to r.
    - Enter the third loop going through each of the inputs. Here, iterate through i from 1 to l.
      - Define a perturbed input $\boldsymbol{u}_p = M(m)P(h)E(i) + \boldsymbol{u}_{ss}$.
      - Simulate the system under the perturbed input $\boldsymbol{u}_p$ at q time-points equally spaced by a difference of Δt, to yield $\boldsymbol{x}^{ihm}(t)$.
      - Additionally define the steady-state of this simulation as $\boldsymbol{x}_{ss}^{ihm}$.
      - Calculate $\mathcal{P}_{\text{Temp}} = \Delta t \left(\boldsymbol{x}^{ihm}(t) - \boldsymbol{x}_{ss}^{ihm}\right)\left(\boldsymbol{x}^{ihm} - \boldsymbol{x}_{ss}^{ihm}\right)^{\intercal}$.
      - Finally, add the contribution to the overall Gramian as

$$\mathcal{P} = \mathcal{P} + \frac{1}{rsM(m)^2}\mathcal{P}_{\text{Temp}}$$

    - End the third loop.
  - End the second loop.
- End the first loop.

- Return the empirical controllability Gramian $\mathcal{P}$.

Now compute the empirical observability Gramian $\mathcal{Q}$ following the approach outlined in Section 1.3:

- Define $\mathcal{Q}$ as an empty $n_L \times n_L$ matrix.
- Define a list of s random input perturbation sizes (M). Here, typically 100 random sizes from 0 to the magnitude of $\boldsymbol{x}_0$ are selected.
- Define a list of r perturbation directions in state-space. Here the algorithm currently employs $r = 2$ corresponding to an increase in all initial conditions and a decrease in all initial conditions. Additionally, define $P(h)$ to represent an $n_L \times n_L$ diagonal matrix of the h-th perturbation direction.
- Define a set of n_L unit vectors (E) corresponding to each of the state-variables.
- Enter the first loop going through each of the perturbation sizes. This loop is parallelized using Matlab's 'parfor' function. Here, iterate through m from 1 to s.
  - Enter the second loop going through each of the perturbation directions. Here, iterate through h from 1 to r.
    * Define a temporary matrix W for storing results from the following loop.
    * Enter the third loop going through each of the inputs. Here, iterate through i from 1 to $\tilde{n}$.
      · Define a perturbed initial condition $\boldsymbol{x}_p = M(m)P(h)E(i) + \boldsymbol{x}_0$.
      · Additionally, recalculate the conserved totals using the perturbed initial conditions (so as to avoid violating conservation).
      · Simulate the system under the undisturbed input $\boldsymbol{u}_{ss}$ with the perturbed initial condition $\boldsymbol{x}_p$ at q time-points equally spaced by a difference of Δt, to yield an output $\boldsymbol{y}^{ihm}(t)$.
      · Additionally define the steady-state of the output of this simulation as $\boldsymbol{y}_{ss}^{ihm}$.
      · Calculate $\boldsymbol{w} = \boldsymbol{y}^{ihm}(t) - \boldsymbol{y}_{ss}^{ihm}$.
      · Concatenate each column of $\boldsymbol{w}$ into the i-th row of W.
    * End the third loop.
    * Calculate $\mathcal{Q}_{\text{Temp}} = WW^{\intercal}$.
    * Finally, add the contribution to the overall Gramian as

$$\mathcal{Q} = \mathcal{Q} + \frac{\Delta t}{rsM(m)^2} P(h)\mathcal{Q}_{\text{Temp}}P(h)^{\intercal}.$$

  - End the second loop.
- End the first loop.
- Return the empirical observability Gramian $\mathcal{Q}$.

Given $\mathcal{P}$ and $\mathcal{Q}$, now compute the balancing transformation as follows:

- Perform a Cholesky factorisation of both of the Gramians using Matlab's inbuilt 'chol' function. This yields $\mathcal{P} = L^{\intercal}L$ and $\mathcal{Q} = R^{\intercal}R$.
- Take a singular value decomposition of the newly formed matrix $LR^{\intercal}$, via Matlab's inbuilt 'svd' function, to obtain $LR^{\intercal} = U\Sigma V^{\intercal}$ with Σ an $n_L \times n_L$ matrix (of the form $\Sigma = \text{diag}(\sigma_1^2, \dots, \sigma_{n_L}^2)$).
- Finally this yields a balancing transformation $T = \Sigma^{-\frac{1}{2}}V^{\intercal}R$ and its inverse $\bar{T} = L^{\intercal}U\Sigma^{-\frac{1}{2}}$.

Now seek to truncate the system one dimension at a time as follows:

- Define new reduced dimension $\tilde{n} < n_L$.
- Calculate the transformed initial conditions $\tilde{\boldsymbol{x}}_0 = T\boldsymbol{x}_0$, such that $\tilde{x}_{0,i} \in \tilde{\boldsymbol{x}}_0$ represents the i-th transformed initial condition.
- Now define reduced variables with residuals $\bar{\boldsymbol{x}}(t) = \left\{\tilde{x}_1(t), \ldots, \tilde{x}_{\tilde{n}}(t), \tilde{x}^*_{0,\tilde{n}+1}, \ldots, \tilde{x}^*_{0,n_L}\right\}$, where $\tilde{x}^*_{0,i}$ represents the initial condition of the i-th truncated, balanced state-variable.
- Hence construct the reduced system as $\dot{\tilde{\boldsymbol{x}}}(t) = PTL\boldsymbol{f}\left(\bar{L}\bar{T}\bar{\boldsymbol{x}}(t)\right)$ via substitution into DiffFuncs, where $P = [I_{\tilde{n}}\ 0]$.
- Simulate this reduced system and compare to the original output (OutO) to obtain a measure of error.

Finally, return the lowest dimensional system that maintains an acceptable maximal relative error bound $\varepsilon < \varepsilon_c$. In the paper this has been set such that $\varepsilon_c = 0.05$.

References

[1] Antoulas, A.C.: Approximation of Large-scale Dynamical Systems. Advances in Design and Control. Society for Industrial and Applied Mathematics, Philadelphia (2005)

[2] Reder, C.: Metabolic control theory: a structural approach. Journal of Theoretical Biology **135**(2), 175–201 (1988)

[3] Hahn, J., Edgar, T.F.: An improved method for nonlinear model reduction using balancing of empirical Gramians. Computers and Chemical Engineering **26**(10), 1379–1397 (2002)

[4] Tindall, M., Porter, S., Wadhams, G., Maini, P., Armitage, J.: Spatiotemporal modelling of CheY complexes in *Escherichia coli* chemotaxis. Progress in Biophysics and Molecular Biology **100**(1), 40–46 (2009)
